# Development of the DONOR prediction model on the risk of hypertensive complications in oocyte donation pregnancy: study protocol for a multicentre cohort study in the Netherlands

**DOI:** 10.1136/bmjopen-2023-079394

**Published:** 2024-07-03

**Authors:** Géraldine C M Lafeber, Vera H W Van der Endt, Yvonne Louwers, Saskia le Cessie, Marie-Louise P van der Hoorn, Eileen E L O Lashley

**Affiliations:** 1 Obstetrics & Gynecology, Leiden University Medical Center, Leiden, The Netherlands; 2 Obstetrics and Gynecology, Erasmus MC, Rotterdam, The Netherlands; 3 Epidemiology, Leids Universitair Medisch Centrum, Leiden, The Netherlands

**Keywords:** Reproductive medicine, Hypertension, Prognosis, Maternal medicine, EPIDEMIOLOGY

## Abstract

**Introduction:**

Oocyte donation (OD) pregnancy is accompanied by a high incidence of hypertensive complications, with serious consequences for mother and child. Optimal care management, involving early recognition, optimisation of suitable treatment options and possibly eventually also prevention, is in high demand. Prediction of patient-specific risk factors for hypertensive complications in OD can provide the basis for this. The current project aims to establish the first prediction model on the risk of hypertensive complications in OD pregnancy.

**Methods and analysis:**

The present study is conducted within the DONation of Oocytes in Reproduction project. For this multicentre cohort study, at least 541 OD pregnancies will be recruited. Baseline characteristics and obstetric data will be collected. Additionally, one sample of maternal peripheral blood and umbilical cord blood after delivery or a saliva sample from the child will be obtained, in order to determine the number of fetal–maternal human leucocyte antigen mismatches. Following data collection, a multivariate logistic regression model will be developed for the binary outcome hypertensive complication ‘yes’ and ‘no’. The Prediction model Risk Of Bias ASsessment Tool will be used as guide to minimise the risk of bias. The study will be reported in line with the ‘Transparent Reporting of a multivariable prediction model for Individual Prognosis Or Diagnosis’ guideline. Discrimination and calibration will be determined to assess model performance. Internal validation will be performed using the bootstrapping method. External validation will be performed with the ‘DONation of Oocytes in Reproduction individual participant data’ dataset.

**Ethics and dissemination:**

This study is approved by the Medical Ethics Committee LDD (Leiden, Den Haag, Delft), with protocol number P16.048 and general assessment registration (ABR) number NL56308.058.16. Further results will be shared through peer-reviewed journals and international conferences.

**Strengths and limitations of this study**

- TRIPOD (Transparent Reporting of a multivariable prediction model for Individual Prognosis Or Diagnosis) and PROBAST (Prediction model Risk Of Bias Assessment Tool) guidelines will be adhered to, ensuring valid methodology and reporting.
- External validation will be performed using available individual participant data from the DONation of Oocytes in Reproduction individual participant data dataset, which will optimise performance evaluation and improve predictive accuracy.
- Assessment of secondary endpoints time of onset and severity of hypertensive complications require larger sample sizes and will therefore be assessed in a later stage.
- The inclusion of a retrospective cohort might introduce bias due to the reliance on medical record, recall bias and incomplete data.
- While ‘fetal–maternal human leucocyte antigen (HLA) (mis)matches’ cannot be used as candidate predictor as non-invasive fetal HLA typing is not yet possible, the individual prognostic value will be assessed.

## Introduction

Subfertility causes a significant socioeconomic burden on global scale. Women facing challenges in generating healthy oocytes for successful pregnancy are posed with the opportunity to conceive through oocyte donation (OD).^1^ In OD, a donor oocyte is used as opposed to an oocyte retrieved from the intended mother.^2 3^ Since its introduction in 1984,^1^ ten thousands of OD procedures have been performed per year worldwide, with high success rates.^4–8^ It is estimated that over 8% of all in vitro fertilisation (IVF) cycles are achieved using donated oocytes in Europe.^7^ Numbers of OD are rising due to a delay in childbirth which leads to increased maternal age and consequently elevated rates of reproductive issues such as ovarian failure. Further, the expansion of indications for OD use, such as reception of oocytes from partner, contributes to the rise of OD numbers.^9^

Despite the promising pregnancy and birth rates, however, OD is associated with serious increased obstetric risks. OD pregnancies show a higher incidence of hypertensive complications, such as pregnancy-induced hypertension (PIH), pre-eclampsia (PE) and haemolysis, elevated liver enzymes and low platelets (HELLP) syndrome and other complications due to placental pathology, compared with naturally conceived and IVF pregnancies.^8 10–15^ The chance of developing hypertensive complications in OD pregnancy is more than doubled when compared with conventional IVF and intracytoplasmic sperm injection and at least tripled when compared with naturally conceived pregnancies.^10 14 16–19^ When translated into absolute risks, the probability of developing hypertensive complications during OD pregnancy ranges from 17% to 18.2% for PIH^14 18^ and from 10.0% to 18% for PE.^16 18 19^

Although the present-day outcome of hypertensive complications in pregnancy is generally good, PE remains one of the leading causes of maternal death when left untreated.^20^ Moreover, hypertensive complications in pregnancy are associated with increased risk of cardiovascular disease and decreased maternal quality of life in the long term.^21–23^ Additionally, severe PE poses increased risk for fetal growth restriction and preterm birth, as a result of placental insufficiency.^8 10 24^

The ability to predict the risk of hypertensive pregnancy complications preconceptionally could potentially aid in disease management and facilitate preconceptional counselling. Further, this contributes to limiting unnecessary exposure of the donor to the risks associated with oocyte retrieval. Subsequently, insight into the risk of developing hypertensive disease during pregnancy might improve early recognition and increase insight into the pathophysiology. This could in turn lead to new prevention and treatment strategies.

Currently, over 100 multivariate prediction models for PE have been developed,^25–27^ though none of the models is developed nor externally validated for OD pregnancies specifically. As in OD pregnancies, a completely allogeneic fetus needs to be tolerated by the mother, these pregnancies differ in essence from natural pregnancy and current predictors might not be applicable in this population. Hence, new predictors such as the genetic differences between mother and child or oocyte donor characteristics could attribute to the prediction of hypertensive complications in OD pregnancies.^11 28 29^ As a result, current prediction models might underperform in women conceived through OD, counter-acting the optimalisation of disease management which is strongly needed in this population group. Therefore, we aim to develop and externally validate a multivariate prediction model on the risk of developing hypertensive complications in women considering OD.

## Study objectives

### Primary objective

The primary objective of the study is to develop a multivariate prediction model for the development of hypertensive complications in OD pregnancy specifically.

### Secondary objectives

Secondary objective is to assess the prognostic effect of individual factors, such as parity, maternal age, fetal–maternal human leucocyte antigen (HLA) mismatches, and donor characteristics such as ethnicity and HLA phenotype, on development of hypertensive complications during pregnancy in women considering OD.

We aim to examine the predictive performances (discrimination, calibration) of our developed multivariate prediction model and to externally validate the model using individual participant data (IPD).^30^

And finally, we aim to explore if it is feasible to develop prediction models for the development of HELLP and early-onset PE and time to development of PE in women considering OD. For this, separate models will be developed not further detailed in this paper.

## Methods and analysis

### Study setting and design

The present study is performed within the DONOR project, on the DONation of Oocytes in Reproduction. This is a multicentre cohort study designed to determine the association between fetal–maternal HLA mismatches and the development of hypertensive complications in pregnancy.^31^ The study will be performed in seven fertility centres in the Netherlands, with the Leiden University Medical Center as coordinating centre.

### Eligibility criteria

Patients with the following criteria are eligible for inclusion:

1. Pregnant or delivered after OD, embryo donation or surrogacy pregnancy. The fertility procedure can be performed in the Netherlands or abroad.
2. Pregnancy duration ≥20 weeks.
3. Visit(ed) one of the participating centres.

Patients who are mentally or legally incapable of signing the informed consent form and patients with known chromosomal abnormalities (such as Turner syndrome) or fetal abnormalities will be excluded.

### Study population and recruitment

In the prospective cohort, patients who visit the gynaecology department of the participating centres will be assessed for eligibility by the attending physician or nurse. Prospective patient recruitment for the DONOR study started at the coordinating centre on 1 September 2016. Recruitment at Erasmus MC has started in 2019. All other centres will start recruitment in November 2023. Prospective patient recruitment is expected to end on 1 January 2026. In the retrospective cohort, patients who underwent successful OD at one of the participating centres between 2004 and the start of the current study will be approached for participation.

Prior to inclusion, all patients will receive written information and an informed consent form, which comprises a request to obtain permission for gathering data from medical records and a sample of maternal peripheral blood and one sample of umbilical cord blood in the prospective cohort and one sample of saliva of the child in the retrospective cohort. Participants are explicitly informed that participation is entirely voluntary and that they retain the right to withdraw at any time without consequences for subsequent care.

In order to optimise inclusion rate and enable representation of the Dutch population that applies to OD, more centres will be approached for collaboration.

### Data collection

For the prospective cohort, maternal, paternal and donor baseline characteristics and obstetric history will be collected at the initial visit. After delivery, obstetric data will be acquired from the patients’ medical records. In the DONOR study, the follow-up period extends from the initial visit until 6 weeks after delivery. Throughout this period, patients will have regular prenatal and postnatal check-ups in the hospital or primary care, which will be documented in medical records by the attending physician or midwife. Only data from participants with a pregnancy duration ≥20 weeks will be used in further analysis.

From the retrospective cohort, maternal baseline and pregnancy characteristics will be collected from the patients’ medical records. All available data will be entered into the data management system Castor.^32^ An overview of patient data that will be collected is shown in table 1.

**Table 1:** Baseline characteristics

Maternal characteristics	*General* Date of birth, country of origin, ethnicity, socioeconomic status, medical history, family history *Obstetric* Number of previous pregnancies, number of previous miscarriages/induced abortions/ectopic pregnancies or pregnancies of unknown location, indication for OD treatment *Laboratory* HLA typing *Oocyte donation procedure* Age at time of embryo transfer, BMI at time of embryo transfer, smoking/drugs/alcohol use
Paternal characteristics	*General* Date of birth, country of origin, ethnicity, medical history, family history *Sperm retrieval* Sperm from partner versus donor, age at time of sperm retrieval, BMI at time of sperm retrieval, smoking/alcohol/drugs use
Oocyte donor characteristics	Age at time of oocyte retrieval, country where oocyte retrieval took place, ethnicity Laboratory* HLA typing
Procedure	Method used for fertilisation (IVF/ICSI), transfer date, country where the transfer took place, transfer in natural or artificial cycle, use of medication (recipient), fresh or cryopreserved transfer of embryo, number of transferred embryos, kind of pregnancy (oocyte donation, embryo donation or surrogate pregnancy)
Pregnancy	Highest systolic and diastolic blood pressure, proteinuria, diagnosis of PIH/PE/HELLP, use of salicylic acid during pregnancy, addition or change of medication during pregnancy
Delivery and neonatal characteristics	Place of birth (hospital vs home), mode of delivery, medication use during labour, gestational age, singleton or multiple, gender, birth weight, live birth (yes/no)

*In case the donor is known and wants to participate, one sample of peripheral blood could be obtained in order to perform HLA typing.

BMI, body mass index; HELLP, haemolysis, elevated liver enzymes and low platelets; HLA, human leucocyte antigen; ICSI, intracytoplasmic sperm injection; IVF, in vitro fertilisation; PE, pre-eclampsia; PIH, pregnancy-induced hypertension.

### Blood and saliva sampling

In the prospective cohort, peripheral maternal blood and umbilical cord blood will be collected for DNA isolation and HLA typing. In the retrospective cohort, peripheral maternal blood and a saliva sample from the child will be obtained. From these samples, DNA will be extracted and HLA typing will be performed for loci HLA-A, HLA-B, HLA-C, HLA-DQ and HLA-DR, using the Reverse Sequence-Specific Oligonucleotides PCR technique.^33^ Additionally, the number of fetal–maternal HLA mismatches will be calculated at the national reference laboratory for histocompatibility testing (Leiden University Medical Center, LUMC), based on discrepancy in the HLA-A, HLA-B, HLA-C, HLA-DR and HLA-DQ. The definition of high number of HLA mismatches is defined as >5 fetal–maternal HLA mismatches.

In case the donor is known and willing to participate, one sample of peripheral blood will be obtained in order to perform HLA typing as well.

### Control of bias

The Prediction model Risk Of Bias Assessment Tool (PROBAST) will be used to minimise the risk of bias.^34^ The PROBAST is developed as a general tool for critical appraisal of prediction model studies and consists of 4 domains (participants, predictors, outcome and analysis) containing 20 signalling questions to minimise risk of bias. In this study, most (18/20) PROBAST criteria will be met. The following two PROBAST criteria need attention to minimise the risk of bias.

- Item 1.2 (Domain 1: participants). It is possible that some participants appear multiple times in our dataset, as some participants will conceive through OD more than once. As repeated measurements of the same participant are not independent, robust standard errors will be calculated to account for this. Additionally, using information from medical records poses a risk of information bias and the retrospective cohort could be prone to response and recall bias, for which caution will be taken.

Furthermore, we aim to implement the prediction model as a tool for preconceptional counselling in women considering OD, whereas development and validation will be performed on a population that is already ≥20 weeks pregnant through OD. We anticipate that this approach will not significantly impact the performance of the prediction model since the majority of covariates remain constant during pregnancy and we expect the time interval between preconceptional counselling and pregnancy to be short.

- Item 2.3 (Domain 2: predictors). The predictive value of fetal–maternal HLA (mis)matches will be assessed, as previous work from our group^28 29 35^ suggests this could be of significance. However, HLA typing of the child cannot be performed before or during pregnancy. As the prediction model is intended to be applied in this exact period, the variable of fetal–maternal mismatches will not be used as a candidate predictor in the model. Instead, the variable will be assessed to determine its individual prognostic value on the risk of developing hypertensive disease. Moreover, proximate predictors such as ‘the expected amount of fetal–maternal mismatches’ based on the donor HLA typing or ‘relation between patient and oocyte donor (familial “yes” or “no”)’ will be used as candidate predictors in our prediction model.

Furthermore, the current study will be reported in line with the Transparent Reporting of a multivariable prediction model for Individual Prognosis Or Diagnosis (TRIPOD) guideline, which aims to improve the reporting of studies developing, validating, or updating a prediction model.^36 37^ Details regarding the control of bias can be found in online supplemental file 1. Finally, any discrepancies between the protocol and the final study will be thoroughly documented. Transparent reporting could help mitigate bias.

10.1136/bmjopen-2023-079394.supp1 Supplementary data

### Sample size calculation

The required sample size was calculated according to the approach of van Smeden *et al* and Riley *et al*.^38–40^ Based on the current absolute risk of hypertensive complications in patients undergoing OD between 10% and 20%,^14 16 18 19^ a number of six candidate predictor parameters, an anticipated model performance of 0.15, a shrinkage factor of 0.90 and a mean absolute prediction error of 0.05 between observed and true outcome probabilities, the required sample size should be at least 541 OD pregnancies.

According to the latest data, approximately 80 successful oocyte or embryo donation procedures are performed yearly in the Netherlands.^41^ In addition, a significant number of Dutch women undergo oocyte or embryo donation procedures in countries outside the Netherlands such as Spain, where the laws and regulations concerning these procedures differ.^42^ For pregnancy check-ups and delivery, these women return to the Netherlands, which poses the opportunity of participation in our study. Currently, the DONOR cohort^31^ consists of 300 OD pregnancies, of which data on fetal–maternal HLA matching is available for 150 pregnancies. To increase inclusion rate and to optimise representation of the Dutch population that applies to OD, also other centres that perform OD are approached for collaboration. The objective is to collect data from approximately 150 additional pregnancies. Assuming that 50% of all eligible patients visiting one of the participating centres will be included in this study and that approximately 15% is lost to follow-up or does not reach pregnancy>20 weeks, it is estimated that the required sample size will be reached within 24 months.

### Study outcomes

The primary outcome measure, hypertensive complication in pregnancy, is defined according to the 2021 International Society for the Study of Hypertension in Pregnancy (ISSHP) classification.^43^ PIH is defined as new onset hypertension with diastolic blood pressure ≥90 mm Hg and/or systolic blood pressure ≥140 mm Hg detected after 20 weeks of gestation. PE is defined as PIH accompanied by one or more of the following: (1) proteinuria, (2) maternal organ dysfunction and (3) uteroplacental dysfunction (fetal growth restriction or abnormal Doppler findings).^22 43^ Superimposed PE is defined by chronic hypertension combined with evidence of uteroplacental dysfunction. Severe hypertension is defined as blood pressure ≥160 mm Hg systolic or ≥110 mm Hg diastolic. HELLP syndrome, which is a serious manifestation of PE, is defined as haemolysis with a microangiopathic blood smear in combination with elevated liver enzymes and a low platelet count.^43^

To enhance generalisability and applicability of a final model, predictor selection should be well defined, standardised and reproducible. In addition, selection of predictors should be based on literature and clinical reasoning. Therefore, the following candidate predictors will be considered^28 44–46^:

1. Fetal–maternal genetical difference, including
  - Fetal–maternal HLA (mis)matches (continuous)
  - Relation (family vs non-family) between patient and oocyte donor (dichotomous)
2. Age recipient (continuous)
3. Nulliparity recipient (dichotomous)
4. Plurality recipient (categorical)
5. Body Mass Index recipient (continuous)
6. Smoking recipient (dichotomous)
7. Natural versus artificial cycle (dichotomous)
8. Ethnicity recipient (categorical)
9. Medical history of recipient related to hypertensive complications in pregnancy (dichotomous)
10. Family history of recipient related to hypertensive complications in pregnancy (dichotomous)
11. Age oocyte donor (continuous)
12. Sperm origin (father vs donor) (dichotomous)
13. Use of medication during pregnancy, specifically acetylsalicylic acid (dichotomous)

### Data analysis

All statistical analyses will be performed using R.

### Model development

A multivariate logistic regression model will be developed for the binary outcome hypertensive complications in pregnancy ‘yes’ and ‘no’. The model will include the preselected candidate predictors.

As part of the secondary objectives, we aim to predict the risk of developing HELLP and time to development of PE. To achieve this, multivariate logistic regression models will be developed for the binary outcomes early PE (≤34 weeks) and HELLP ‘yes’ or ‘no’.^43^ Additionally, in order to predict the time of development of PE, a multivariate linear regression model will be developed for the continuous outcome gestation age at development of PE, with a minimum of 20 weeks and a maximum of 42 weeks. Possible non-linear associations between the continuous predictors and the outcome will be examined, using spline models. Potential multiplicative interaction will be explored by adding interaction terms to the model, and use of Lasso regression for variable selection will be considered.^47^

### Model performance

Internal validation will be performed to assess model performance, and discrimination and calibration will be determined. Discrimination describes the ability of the model to discriminate between events and non-events and will be evaluated with the area under the receiver curve. Calibration describes the relation between the observed risks within the population and the predicted risks and will be assessed by calibration plots, plotting the relation between the observed and predicted risks.^48^ Internal validation will be performed using the bootstrapping method with 100 bootstrap samples. The calibration slope from the bootstrapping procedure will be used as uniform shrinkage factor to calculate new regression coefficients to prevent overfitting.^47^ External validation will be performed using the ‘DONation of Oocytes in Reproduction individual participant data’ (DONOR IPD). The DONOR IPD database is currently being set up and will include at least 2301 women pregnant after OD and beyond 20 weeks of gestation.^30^ In this study original data from multiple studies will be combined into a single database is therefore suitable for model performance assessment and external validations.^49 50^

### Handling of missing data

In case the amount of missing data in some of the predictors is substantial and cannot be ignored, a multiple imputation method will be applied.^51 52^

### Patient and public involvement

During the development of the study protocol, Freya was consulted for input and advice. Freya is the Dutch society for patients with fertility problems and many patients who apply to OD are members of the association. Study information will be published on their website, and information on progress and results will be presented to patients during meetings, patient days and webinars.

## Discussion

Each year, ten thousands of OD procedures are performed successfully worldwide.^4–7^

Although the numbers are rising due to delay of maternal age and increasing indications for OD, the method is accompanied by a high incidence of complications, in particular hypertensive disorders of pregnancy. The possibility to predict these complications could potentially aid in disease management and even prevention, yet current prognostic factors and prediction models are not developed, nor validated, for the OD population. With this project, we propose to develop the first prediction model on the risk of hypertensive complications in pregnancy in women considering OD. In clinical practice, clinicians could use this developed prediction model to more accurately classify the woman as low or high risk for hypertensive complications in pregnancy, allowing for more personalised counselling for women considering OD. As a consequence, this might facilitate in decision making for the patient. In the case, the woman is already pregnant, knowledge of hypertensive complications risk might aid the clinician in early recognition of the disease and risk stratification during pregnancy, resulting in better healthcare management.

One fundamental strength of this project is that reporting will be in line with the TRIPOD guideline, aiming to reduce bias by enhancing transparency with regard to development, validation and updates of our model.^34 36^ Additionally, most PROBAST criteria will be met in this study, further mitigating bias.

Besides, available IPD^30^ makes external validation possible. This will enable optimal performance evaluation and improve predictive accuracy, as populations independent of that used to develop the model will be used with different environmental, geographical and socioeconomic characteristics and different times of conception.^30 37^

It should be acknowledged that control of bias may be impeded as most data will be acquired from medical records. The prediction model will be developed and validated in a population that is already pregnant after OD, whereas the model is intended for facilitating preconceptional counselling. Although differences are expected to be small and insignificant, the prediction model might overperform or underperform in the intended population group. Therefore, external validation in a population of women considering OD is warranted.

Furthermore, the present project also includes secondary objectives of predicting the severity of PE and its time of onset. The risk of developing severe PE is lower than the risk of hypertensive complication in OD. Additionally, the prediction of time onset of PE is only possible in patients who actually developed PE. Larger sample sizes are thus required for these objectives. Accordingly, development of these models will be undertaken during a subsequent phase.

Lastly, the predictor ‘fetal–maternal HLA (mis)matches’ cannot be used as a candidate predictor in the model as it is unknown at time of proposed use of the model. The variable will be assessed to determine its individual prognostic value on the risk of developing hypertensive disease. In addition, the expected number of fetal–maternal mismatches could be calculated based on donor and paternal HLA profiles. Still, the DONOR IPD dataset does not yet contain data on HLA genotyping, thus the predictive value of fetal–maternal HLA (mis)matches cannot be validated externally. To enable external validation in future, development of a database including information on fetal and maternal HLA genotypes is required. We are currently working on a non-invasive method to perform antenatal HLA typing and calculation of mismatches. This will facilitate including the variable in future models and improve the accuracy of our predictions. Additionally, future studies should carefully consider the financial implications of HLA typing as this may impact the feasibility of large-scale implementation and clinical practice. To conclude, this study aims to develop and externally validate the first prediction model on the risk of hypertensive complications in pregnancy in women considering OD. The possibility of predicting the risk of hypertensive complications in pregnancy could potentially aid in disease management of women considering OD or in women pregnant after OD.

## Ethics and dissemination

The study will be conducted according to the principles of the Declaration of Helsinki. All patients will obtain written information of the study and in case of participation, the informed consent form should be signed prior to inclusion. This study is approved by the Medical Ethical Committee LDD (Leiden, Den Haag, Delft), with protocol number P16.048 and general assessment registration (ABR) number NL56308.058.16. All clinical data and biomaterial are pseudonymised by assigning a unique code and will be collected in the data management system Castor. Only a minimum number of members of our project group will have access to this database. The results of our study will be disseminated via publications in gynaecology-related journals. Further, the results of our study might be presented at (international) conferences.

## Supplementary Material

Reviewer comments

Author's manuscript
