# Supplementary material for: Development of the DONOR prediction model on the risk of hypertensive complications in oocyte donation pregnancy: study protocol for a multicentre cohort study in the Netherlands
Source: BMJ Open. 2024 Jul 3;14(7):e079394. doi: 10.1136/bmjopen-2023-079394 (PMC11227773; doi:10.1136/bmjopen-2023-079394)
Supplement: Author's manuscript [file bmjopen-2023-079394.draft_revisions.pdf]

BMJ Open is committed to open peer review. As part of this commitment we make the peer review history of every article we publish publicly available.

When an article is published we post the peer reviewers' comments and the authors' responses online. We also post the versions of the paper that were used during peer review. These are the versions that the peer review comments apply to.

The versions of the paper that follow are the versions that were submitted during the peer review process. They are not the versions of record or the final published versions. They should not be cited or distributed as the published version of this manuscript.

BMJ Open is an open access journal and the full, final, typeset and author-corrected version of record of the manuscript is available on our site with no access controls, subscription charges or pay-per-view fees (http://bmjopen.bmj.com).

If you have any questions on BMJ Open's open peer review process please email info.bmjopen@bmj.com

BMJ Open

Development of the DONOR prediction model on the risk of hypertensive complications in oocyte donation pregnancy: study protocol for a prospective and retrospective multicenter cohort study

Journal:	*BMJ Open*
Manuscript ID	bmjopen-2023-079394
Article Type:	Protocol
Date Submitted by the Author:	30-Aug-2023
Complete List of Authors:	Lafeber, Géraldine; Leiden University Medical Center, Obstetrics & Gynecology<br>Van der Endt, Vera H.W.; Leiden University Medical Center, Obstetrics & Gynaecology<br>Louwers, Yvonne; Erasmus MC, Obstetrics and Gynecology<br>le Cessie, S; Leids Universitair Medisch Centrum, Epidemiology<br>van der Hoorn, Marie-Louise; Leids Universitair Medisch Centrum, Obstetrics & Gynaecology<br>Lashley, Eileen; Leids Universitair Medisch Centrum, Obstetrics & Gynaecology
Keywords:	Reproductive medicine < GYNAECOLOGY, Hypertension < CARDIOLOGY, Prognosis, Maternal medicine < OBSTETRICS, EPIDEMIOLOGY

SCHOLARONE™
Manuscripts

Development of the DONOR prediction model on the risk of hypertensive complications in oocyte donation pregnancy: study protocol for a prospective and retrospective multicenter cohort study

Géraldine C.M. Lafeber[1*], Vera H. W. van der Endt[1*], Yvonne V Louwers[3], S. le Cessie[2], Marie-Louise P. van der Hoorn[1], Eileen E.L.O. Lashley[1]

[1] Department of Obstetrics and Gynecology, Leiden University Medical Center, Leiden, the Netherlands
[2] Department of Clinical Epidemiology, Leiden University Medical Center, Leiden, the Netherlands
[3] Department of Obstetrics and Gynecology, Erasmus Medical Center, the Netherlands

* Authors contributed equally to this manuscript

Keywords
Reproductive medicine, hypertension, prognosis, maternal medicine, epidemiology

Word count
3461

For peer review only - http://bmjopen.bmj.com/site/about/guidelines.xhtml

Abstract

Introduction

Oocyte donation (OD) pregnancy is accompanied with a high incidence of hypertensive complications, with serious consequences for mother and child. Optimal care management, involving early recognition, optimization of suitable treatment options, and possibly eventually also prevention, is of high demand. Prediction of patient specific risk factors for hypertensive complications in OD can provide the basis for this. The current project aims to establish the first prediction model on the risk of hypertensive complications in OD pregnancy.

Methods and analysis

The present study is conducted within the DONation of Oocytes in Reproduction (DONOR) project. For this prospective and retrospective, multicenter cohort study, at least 541 OD pregnancies will be recruited. Baseline characteristics and obstetric data will be collected. Additionally, one sample of maternal peripheral blood and umbilical cord blood after delivery or a saliva sample from the child will be obtained, in order to determine the number of fetal-maternal HLA-mismatches. Following data collection, a multivariate logistic regression model will be developed for the binary outcome hypertensive complication 'yes' and 'no'. The Prediction model Risk Of Bias ASsessment Tool (PROBAST) will be used as guide to minimize the risk of bias. The study will be performed and reported in line with the 'Transparent Reporting of a multivariable prediction model for Individual Prognosis Or Diagnosis (TRIPOD)' guideline. Discrimination and calibration will be determined to assess model performance. Internal validation will be performed using the bootstrapping method. External validation will be performed with the 'DONation of Oocytes in Reproduction individual participant data' (DONOR IPD) dataset.

Ethics and dissemination

This study is approved by the Medical Ethics Committee LDD (Leiden, Den Haag, Delft). Further, results will be shared through peer-reviewed journals and international conferences (P16.048, ABR NL56308.058.16).

Article summary

Strengths and limitations of this study:

- This study aims to develop and externally validate the first prediction model on the risk of hypertensive complication during OD pregnancy.
- External validation will be performed using available individual participant data from the DONOR IPD dataset, which will optimize performance evaluation and improve predictive accuracy.
- TRIPOD and PROBAST guidelines will be adhered to, ensuring valid methodology and reporting.
- The inclusion of a retrospective cohort might introduce bias due to the reliance on medical record, recall bias and incomplete data.
- While 'fetal-maternal HLA (mis)matches' cannot be used as candidate predictor as non-invasive fetal HLA-typing is not yet possible, the individual prognostic value will be assessed.

For peer review only - http://bmjopen.bmj.com/site/about/guidelines.xhtml

For peer review only

Introduction

Subfertility causes a significant socioeconomic burden on global scale. Women unable to produce viable oocytes for healthy offspring are posed the opportunity to conceive through oocyte donation (OD)[1]. In OD a donor oocyte is used as opposed to an oocyte retrieved from the intended mother[2, 3]. Since its introduction in 1984[1], ten thousands of OD procedures have been performed per year worldwide, with high success rates[4-8]. It is estimated that over 8% of all IVF cycles are achieved using donated oocytes in Europe[7]. Numbers of OD are rising due to a delay in childbirth which leads to increased maternal age and consequently elevated rates of reproductive issues such as ovarian failure. Further, the expansion of indications for OD use, such as shared lesbian motherhood or reception of oocytes from partner (ROPA) contribute to the rise of OD numbers [9].

Despite the promising pregnancy and birth rates, however, OD is associated with serious increased obstetric risks. OD pregnancies show a higher incidence of hypertensive complications, such as pregnancy induced hypertension (PIH), preeclampsia (PE) and hemolysis, elevated liver enzymes and low platelets (HELLP) syndrome and other complications due to placental pathology, compared to naturally conceived and IVF pregnancies [8, 10-15]. The chance of developing hypertensive complications in OD pregnancy is more than doubled when compared to conventional IVF and intracytoplasmic sperm injection (ICSI) and at least tripled when compared to naturally conceived pregnancies[10, 14, 16-19]. When translated into absolute risks, the probability of developing hypertensive complications during OD pregnancy ranges from 17% to 18.2% for PIH[14, 18] and from 10.0% to 18% for PE[16, 18, 19].

Although the present-day outcome of hypertensive complications in pregnancy is generally good, PE remains one of the leading causes of maternal death when left untreated [20]. Moreover, hypertensive complications in pregnancy are associated with increased risk of cardiovascular disease and decreased maternal quality of life on the long term [21-23]. Additionally, severe PE poses increased risk for fetal growth restriction and preterm birth, as a result of placental insufficiency [8, 10, 24].

The ability to predict the risk of hypertensive pregnancy complications pre-conceptionally, could potentially aid in disease management and facilitate pre-conceptional counseling. Further, this contributes to limiting unnecessary exposure of the donor to the risks associated with oocyte retrieval. Subsequently, insight in the risk of developing hypertensive disease during pregnancy might improve early recognition and increase insight in the pathophysiology. This could in turn lead to new prevention- and treatment strategies.

Currently, over 100 multivariate prediction models for PE have been developed[25-27], though none of the models is developed nor externally validated for OD pregnancies specifically. As in OD pregnancies a completely allogeneic fetus needs to be tolerated by the mother, these pregnancies differ in essence from natural pregnancy and current predictors might not be applicable in this population. Hence, new predictors such as the genetic differences between mother and child or oocyte donor characteristics, could attribute to the prediction of hypertensive complications in OD pregnancies[11, 28, 29]. As a result, current prediction models might underperform in women conceived through OD, counter-acting the optimalisation of disease management which is strongly needed in this population group. Therefore, we aim to develop and externally validate a multivariate prediction model on the risk of developing hypertensive complications in women considering OD.

For peer review only

For peer review only - http://bmjopen.bmj.com/site/about/guidelines.xhtml

Study objectives

Primary objective

The primary objective of the study is to develop a multivariate prediction model for the development of hypertensive complications in OD pregnancy specifically.

Secondary objectives

Secondary objective is to assess the prognostic effect of individual factors, such as parity, maternal age, fetal-maternal HLA-mismatches, and donor characteristics such as ethnicity and HLA-phenotype, on development of hypertensive complications during pregnancy in women considering OD.

We aim to examine the predictive performances (discrimination, calibration) of our developed multivariate prediction model and to externally validate the model using individual participant data (IPD)[30].

And finally, we aim to explore if it is feasible to develop prediction models for the development of severe PE(including HELLP and early-onset preeclampsia) and time to development of preeclampsia in women considering OD. For this, separate models will be developed not further detailed in this paper.

For peer review only

For peer review only - http://bmjopen.bmj.com/site/about/guidelines.xhtml

Methods and Analysis

Study design

The present study is performed within the DONOR project, on the DONation of Oocytes in Reproduction. This is a multicenter prospective and retrospective cohort study designed to determine the association between fetal-maternal HLA-mismatches and the development of hypertensive complications in pregnancy [31].

Eligibility criteria

Patients with the following criteria are eligible for inclusion:

1) Pregnant or delivered after oocyte donation, embryo donation or surrogacy pregnancy. The fertility procedure can be performed in the Netherlands or abroad.
2) Pregnancy duration ≥ 20 weeks.
3) Visit(ed) one of the participating centers.

Patients who are mentally or legally incapable of signing the informed consent form and patients with known chromosomal abnormalities (such as Turner syndrome) or fetal abnormalities will be excluded.

Study population and recruitment

In the prospective cohort, patients who visit the gynecology department of the participating centers will be assessed for eligibility by the attending physician or nurse. Prospective patient recruitment for the DONOR study started at the coordinating center on 1 September 2016. Recruitment at Erasmus MC (EMC) has started in 2019. All other centers will start recruitment in November 2023. In the retrospective cohort, patients who underwent successful OD at one of the participating centers between 2004 and the start of the current study will be approached for participation.

Prior to inclusion, all patients will receive written information and an informed consent form, which comprises a request to obtain permission for gathering data from medical records and a sample of maternal peripheral blood and one sample of umbilical cord blood in the prospective cohort and one sample of saliva of the child in the retrospective cohort. Participants are explicitly informed that participation is entirely voluntary and that they retain the right to withdraw at any time without consequences for subsequent care.

In order to optimize inclusion rate and enabling representation of the Dutch population that applies to OD, more centers will be approached for collaboration.

Data collection

For the prospective cohort, maternal, paternal and donor baseline characteristics and obstetric history will be collected at the initial visit. After delivery, obstetric data will be acquired from the patients' medical records. In the DONOR study, the follow-up period extends from the initial visit until six weeks after delivery. Throughout this period, patients will have regular prenatal and postnatal check-ups in the hospital or primary care, which will be documented in medical records by the attending physician or midwife. Only data from participants with a pregnancy duration ≥20 weeks, will be used in further analysis.

For peer review only - http://bmjopen.bmj.com/site/about/guidelines.xhtml

From the retrospective cohort, maternal baseline and pregnancy characteristics will be collected from the patients' medical records. All available data will be entered into the data management system Castor[32]. An overview of patient data that will be collected is shown in **Table 1**.

Blood and saliva sampling

In the prospective cohort, peripheral maternal blood and umbilical cord blood will be collected for DNA isolation and HLA typing. In the retrospective cohort, peripheral maternal blood and a saliva sample from the child will be obtained. From these samples, DNA will be extracted and HLA-typing will be performed for loci HLA-A, HLA-B, HLA-C, HLA-DQ and HLA-DR, using Reverse Sequence Specific Oligonucleotides PCR technique [33]. Additionally, the number of fetal-maternal HLA-mismatches will be calculated at the national reference laboratory for histocompatibility testing (Leiden University Medical Center, LUMC), based on discrepancy on the HLA-A, -B, -C, -DR and -DQ. The definition of high number of HLA-mismatches is defined as >5 fetal-maternal HLA-mismatches. In case the donor is known and willing to participate, one sample of peripheral blood will be obtained in order to perform HLA-typing as well.

Control of bias

The current study will be performed and reported on in line with the Transparent Reporting of a multivariable prediction model for Individual Prognosis Or Diagnosis (TRIPOD) guideline, which aims to improve the reporting of studies developing, validating, or updating a prediction model[34, 35]. Moreover, the Prediction model Risk Of Bias Assessment Tool (PROBAST) will be used to minimize the risk of bias [36]. The PROBAST is developed as a general tool for critical appraisal of prediction model studies and consists of 4 domains (participants, predictors, outcome and analysis) containing 20 signaling questions to minimalize risk of bias. In this study most (18/20) PROBAST criteria will be met. The following two PROBAST criteria need attention to minimize the risk of bias.

- Item 1.2 (Domain 1: participants). It is possible that some participants appear multiple times in our dataset, as some participants will conceive through OD more than once. As repeated measurements of the same participant are not independent, robust standard errors will be calculated to account for this. Additionally, using information from medical records poses a risk of information bias and the retrospective cohort could be prone to response and recall bias, for which caution will be taken. Furthermore, we aim to implement the prediction model as a tool for pre-conceptional counseling in women considering OD, whereas development and validation will be performed on a population that is already ≥20 weeks pregnant through OD. We anticipate that this approach will not significantly impact the performance of the prediction model since the majority of covariates remains constant during pregnancy and we expect the time interval between pre-conceptional counseling and pregnancy to be short.
- Item 2.3 (Domain 2: predictors). The predictive value of fetal-maternal HLA-(mis)matches will be assessed, as previous work from our group [28, 29, 37] suggests this could be of significance. However, HLA-typing of the child cannot be performed before or during pregnancy. As the prediction model is intended to be applied in this exact period, the variable of fetal-maternal mismatches will not be used as a candidate predictor in the model. Instead, the variable will be assessed to determine its individual prognostic value on the risk of developing hypertensive disease. Moreover, proximate predictors such as 'the expected amount of fetal-maternal mismatches' based upon the donor HLA typing or 'relation between patient and oocyte donor (familial 'yes' or 'no')' will be used as candidate predictors in our prediction model.

For peer review only - http://bmjopen.bmj.com/site/about/guidelines.xhtml

Sample size calculation

The required sample size was calculated according to the approach of Van Smeden et al. (2019) and Riley et al. (2019, 2020) [38-40]. Based on the current absolute risk of hypertensive complications in patients undergoing OD between 10-20%[14, 16, 18, 19], a number of six candidate predictor parameters, an anticipated model performance of 0.15, a shrinkage factor of 0.90, and a mean absolute prediction error of 0.05 between observed and true outcome probabilities, the required sample size should be at least 541 OD pregnancies.

According to the latest data, approximately 80 successful oocyte or embryo donation procedures are performed yearly in the Netherlands[41]. In addition, a significant number of Dutch women undergo oocyte or embryo donation procedures in countries outside The Netherlands such as Spain, where the laws and regulations concerning these procedures differ[42]. For pregnancy check-ups and delivery, these women return to the Netherlands, which poses the opportunity of participation in our study. Currently, the DONOR cohort [31] consists of 300 OD pregnancies, of which data on fetal-maternal HLA-matching is available for 150 pregnancies. To increase inclusion rate and to optimize representation of the Dutch population that applies to OD, also other centers that perform OD are approached for collaboration. The objective is to collect data from approximately 150 additional pregnancies. Assuming that 50% of all eligible patients visiting one of the participating centers will be included in this study, and that approximately 15% is lost to follow-up or does not reach pregnancy > 20 weeks, it is estimated that the required sample size will be reached within 24 months.

Study outcomes

The primary outcome measure, hypertensive complication in pregnancy, is defined according to the ISSHP classification [43, 44]. PIH is defined as new onset hypertension with diastolic blood pressure ≥90 mmHg and/or systolic blood pressure ≥140 mmHg detected after 20 weeks of gestation. PE is defined as PIH accompanied by one or more of the following: 1) proteinuria, 2) maternal organ dysfunction, 3) uteroplacental dysfunction (fetal growth restriction or abnormal doppler findings)[22]. Severe PE is defined as PE with blood pressure ≥160 mmHg systolic or ≥110 mmHg diastolic, in the presence of HELLP syndrome or in early-onset[44]. HELLP syndrome is defined as hemolysis with a microangiopathic blood smear in combination with elevated liver enzymes and a low platelet count[43]. Early-onset PE is defined as PE occurring before 34 weeks of gestation[44].

Based on literature review and clinical reasoning, the following candidate predictors will be considered[28, 45-47]:

1) Fetal-maternal genetical difference, including
    - Fetal-maternal HLA-(mis)matches (continuous)
    - Relation (family vs non-family) between patient and oocyte donor (dichotomous)
2) Age recipient (continuous)
3) Nulliparity recipient (dichotomous)
4) Plurality recipient (categorical)
5) BMI recipient (continuous)
6) Smoking recipient (dichotomous)
7) Natural vs artificial cycle (dichotomous)
8) Ethnicity recipient (categorical)
9) Medical history of recipient related to hypertensive complications in pregnancy (dichotomous)

For peer review only - http://bmjopen.bmj.com/site/about/guidelines.xhtml

10) Family history of recipient related to hypertensive complications in pregnancy (dichotomous)
11) Age oocyte donor (continuous)
12) Sperm origin (father vs donor) (dichotomous)

Data analysis

All statistical analyses will be performed using R.

Model development

A multivariate logistic regression model will be developed for the binary outcome hypertensive complications in pregnancy 'yes' and 'no'. The model will include the preselected candidate predictors.

As part of the secondary objectives, we aim to predict the risk on developing severe PE and time to development of PE. To achieve this, a multivariate logistic regression model will be developed for the binary outcome severe PE 'yes' or 'no'. Severe PE is defined as blood pressure ≥160 mmHg systolic or ≥110 mmHg diastolic, the presence of HELLP (hemolysis, elevated liver enzymes and low platelets) syndrome or onset of PE before 34 weeks of gestation)[43, 44]. Additionally, in order to predict the time of development of PE, a multivariate linear regression model will be developed for the continuous outcome gestation age at development of PE, with minimum of 20 weeks and maximum of 42 weeks)'.Possible non-linear associations between the continuous predictors and the outcome will be examined, using spline models. Potential multiplicative interaction will be explored by adding interaction terms to the model, and use Lasso regression for variable selection will be considered[48].

Model performance

Internal validation will be performed to assess model performance, discrimination and calibration will be determined. Discrimination describes the ability of the model to discriminate between events and non-events and will be evaluated with the area under the receiver curve (AUC). Calibration describes the relation between the observed risks within the population and the predicted risks and will be assessed by calibration plots, plotting the relation between the observed and predicted risks[49]. Internal validation will be performed using the bootstrapping method with 100 bootstrap samples. The calibration slope from the bootstrapping procedure will be used as uniform shrinkage factor to calculate new regression coefficients to prevent overfitting[48]. External validation will be performed using the 'DONation of Oocytes in Reproduction individual participant data' (DONOR IPD). The DONOR IPD database is currently being set up and will include at least 2301 women pregnant after OD and beyond 20 weeks of gestation[30]. In this study original data from multiple studies will be combined into a single database is therefore suitable for model performance assessment and external validations[50, 51].

Handling of missing data

In case the amount of missing data in some of the predictors is substantial and cannot be ignored, a multiple imputation method will be applied[52, 53].

For peer review only - http://bmjopen.bmj.com/site/about/guidelines.xhtml

Discussion

Each year, ten thousands of OD procedures are performed successfully worldwide [4-7]. Although the numbers are rising due to delay of maternal age and increasing indications for OD, the method is accompanied with a high incidence of complications, in particular hypertensive disorders of pregnancy. The possibility to predict these complications could potentially aid in disease management and even prevention, yet current prognostic factors and prediction models are not developed, nor validated, for the OD population. With this project, we propose to develop the first prediction model on the risk of hypertensive complications in pregnancy in women considering OD. In clinical practice clinicians could use this developed prediction model to more accurately classify the woman as low or high risk for hypertensive complications in pregnancy, allowing for more personalized counseling for women considering OD. As a consequence this might facilitate in decision making for the patient. In the case the woman is already pregnant, knowledge on hypertensive complications risk might aid the clinician in early recognition of the disease and risk stratification during pregnancy, resulting in better health care management.

One fundamental strength of this project is the adherence to the TRIPOD and PROBAST guidelines, ensuring that essential steps in prediction model development and validation will be performed as recommended [34, 36]. Besides, available IPD [30] makes external validation possible. This will enable optimal performance evaluation and improve predictive accuracy, as populations independent of that used to develop the model will be used with different environmental, geographical, socioeconomic characteristics and different times of conception [30, 35].

It should be acknowledged that control of bias may be impeded as most data will be acquired from medical records. The prediction model will be developed and validated in a population that is already pregnant after OD, whereas the model is intended for facilitating pre-conceptional counseling. Although differences are expected to be small and insignificant, the prediction model might over- or underperform in the intended population group. Therefore, external validation in a population of women considering OD is warranted.

Furthermore, the present project also includes secondary objectives of predicting the severity of PE and its time of onset. The risk of developing severe PE is lower than the risk of hypertensive complication in OD. Additionally, the prediction of time onset of preeclampsia is only possible in patients that actually developed preeclampsia. Larger sample sizes are thus required for these objectives. Accordingly, development of these models will be undertaken during a subsequent phase.

Lastly, the predictor 'fetal-maternal HLA-(mis)matches' cannot be used as a candidate predictor in the model as it is unknown at time of proposed use of the model. The variable will be assessed to determine its individual prognostic value on the risk of developing hypertensive disease. In addition, the expected number of fetal-maternal mismatches could be calculated based on donor and paternal HLA-profiles. Still, the DONOR IPD dataset does not yet contain data on HLA-genotyping, thus the predictive value of fetal-maternal HLA-(mis)matches cannot be validated externally. To enable external validation in future, development of a database including information on fetal and maternal HLA-genotypes is required. We are currently working on a non-invasive method to perform antenatal HLA typing and calculation of mismatches. This will facilitate including the variable in future models and improve the accuracy of our predictions.

To conclude, this study aims to develop and externally validate the first prediction model on the risk of hypertensive complications in pregnancy in women considering OD. The possibility to predict the

For peer review only - http://bmjopen.bmj.com/site/about/guidelines.xhtml

343 risk of hypertensive complications in pregnancy could potentially aid in disease management of
344 women considering OD or in women pregnant after OD.

For peer review only

For peer review only - http://bmjopen.bmj.com/site/about/guidelines.xhtml

Ethics and Dissemination

The study will be conducted according to the principles of the Declaration of Helsinki. All patients will obtain written information of the study and in case of participation, the informed consent form should be signed prior to inclusion. This study is approved by the Medical Ethical Committee of the Leiden University Medical Center (METC LUMC-LDD; P16.048). All clinical data and biomaterial is pseudonymised by assigning a unique code and will be collected in the data management system Castor. Only a minimum number of members of our project group will have access to this database. The results of our study will be disseminated via publications in gynecology related journals. Further, the results of our study might be presented at (international) conferences.

Ethics statements

Patient consent for publication: not applicable.

For peer review only

For peer review only - http://bmjopen.bmj.com/site/about/guidelines.xhtml

Abbreviations

AUC	Area Under the Curve
DONOR	DONation of Oocytes in Reproduction
HELLP	Heymolysis, Elevated Liver Enzymes and Low Platelets
HLA	Human Leukocyte Antigen
ICSI	Intracytoplasmic Sperm Injection
IPD	Individual Participant Data
IVF	In Vitro Fertilization
LUMC	Leiden University Medical Center
METC	Medical Ethics Committee
NC	Naturally conceived
PE	Preeclampsia
PROBAST	Prediction model Risk Of Bias Assessment Tool
ROB	Risk of Bias
TRIPOD	Transparent Reporting of a multivariable prediction model for Individual Prognosis Or Diagnosis

For peer review only

For peer review only - http://bmjopen.bmj.com/site/about/guidelines.xhtml

References

1. Lutjen P, Trounson A, Leeton J, et al. The establishment and maintenance of pregnancy using in vitro fertilization and embryo donation in a patient with primary ovarian failure. Nature. 1984;307(5947):174-5. doi: 10.1038/307174a0
2. Nederlandse Vereniging voor Klinische Embryologie (KLEM). Modelreglement Embryowet [online]. 2018. https://www.nvog.nl/wp-content/uploads/2020/10/Modelreglement-Embryowet-NVOG-en-KLEM-definitief-augustus-2018-aanpassing-15-oktober-2020.pdf
3. Nederlandse Vereniging voor Obstetrie en Gynaecologie (NVOG). STANDPUNT - Geassisteerde voortplanting met gedoneerde gameten en gedoneerde embryo's en draagmoederschap [online]. 2016. https://www.nvog.nl/wp-content/uploads/2020/06/Standpunt-Geassisteerde-voortplanting-met-gedoneerde-gameten-en-gedoneerde-embryo-juni-2016-addendum-12-6-2020.pdf.
4. Society For Assisted Reproductive Technology. National Summary Report [online]. 2018. https://www.sartcorsonline.com/rptCSR_PublicMultYear.aspx?reportingYear=2018.
5. Hogan RG, Wang AY, Li Z, et al. Oocyte donor age has a significant impact on oocyte recipients' cumulative live-birth rate: a population-based cohort study. Fertil Steril. 2019;112(4):724-30. doi: 10.1016/j.fertnstert.2019.05.012
6. Remohí J, Gartner B, Gallardo E, et al. Pregnancy and birth rates after oocyte donation. Fertil Steril. 1997;67(4):717-23. doi: 10.1016/s0015-0282(97)81372-6
7. Wyns C, De Geyter C, Calhaz-Jorge C, et al. ART in Europe, 2018: results generated from European registries by ESHRE†. Human Reproduction Open. 2022;2022(3). doi: 10.1093/hropen/hoac022
8. Savasi VM, Mandia L, Laoreti A, et al. Maternal and fetal outcomes in oocyte donation pregnancies. Hum Reprod Update. 2016;22(5):620-33. doi: 10.1093/humupd/dmw012
9. Bodri D, Nair S, Gill A et al. Shared motherhood IVF: high quality rates in a large study of treatments for lesbian couples using partner donated eggs. Reprod Biomed Online. 2018;36:130-6. doi: 10.1016/j.rbmo.2017.11.006
10. Storgaard M, Loft A, Bergh C, et al. Obstetric and neonatal complications in pregnancies conceived after oocyte donation: a systematic review and meta-analysis. Bjog. 2017;124(4):561-72. doi: 10.1111/1471-0528.14257
11. Van der Hoorn MP, van Egmond A, Swings G, et al. Differential immunoregulation in successful oocyte donation pregnancies compared with naturally conceived pregnancies. J Reprod Immunol. 2014;101-102:96-103. doi: 10.1016/j.jri.2013.08.002
12. Berntsen S, Larsen EC, la Cour Freiesleben N, et al. Pregnancy outcomes following oocyte donation. Best Pract Res Clin Obstet Gynaecol. 2021;70:81-91. doi: 10.1016/j.bpobgyn.2020.07.008
13. Berntsen S, Söderström-Anttila V, Wennerholm UB, et al. The health of children conceived by ART: 'the chicken or the egg?'. Hum Reprod Update. 2019;25(2):137-58. doi: 10.1093/humupd/dmz001
14. Masoudian P, Nasr A, de Nanassy J, et al. Oocyte donation pregnancies and the risk of preeclampsia or gestational hypertension: a systematic review and metaanalysis. Am J Obstet Gynecol. 2016;214(3):328-39. doi: 10.1016/j.ajog.2015.11.020

For peer review only - http://bmjopen.bmj.com/site/about/guidelines.xhtml

15. Levron Y, Dviri M, Segol I, et al. The 'immunologic theory' of preeclampsia revisited: a lesson from donor oocyte gestations. Am J Obstet Gynecol. 2014;211(4):383.e1-5. doi: 10.1016/j.ajog.2014.03.044
16. Blázquez A, García D, Rodríguez A, et al. Is oocyte donation a risk factor for preeclampsia? A systematic review and meta-analysis. J Assist Reprod Genet. 2016;33(7):855-63. doi: 10.1007/s10815-016-0701-9.
17. Moreno-Sepulveda J, Checa MA. Risk of adverse perinatal outcomes after oocyte donation: a systematic review and meta-analysis. J Assist Reprod Genet. 2019;36(10):2017-37. doi: 10.1007/s10815-019-01552-4
18. Jeve YB, Potdar N, Opoku A, et al. Donor oocyte conception and pregnancy complications: a systematic review and meta-analysis. Bjog. 2016;123(9):1471-80. doi: 10.1111/1471-0528.13910
19. Keukens A, van Wely M, van der Meulen C, et al. Pre-eclampsia in pregnancies resulting from oocyte donation, natural conception or IVF: a systematic review and meta-analysis. Hum Reprod. 2022;37(3):586-99. doi: 10.1093/humrep/deab267
20. World Health Organization. Maternal mortality: Key facts [online]. 2021. https://www.who.int/news-room/fact-sheets/detail/maternal-mortality
21. National Institute for Health and Care Excellence (UK): Clinical Guidelines. Hypertension in pregnancy: diagnosis and management [online]. 2019. https://www.nice.org.uk/guidance/ng133
22. Mol BWJ, Roberts CT, Thangaratinam S, et al. Pre-eclampsia. Lancet. 2016;387(10022):999-1011. doi: 10.1016/s0140-6736(15)00070-7
23. Patrícia Medeiros Falcão K, Pedrozo Campos Antunes T, do Nascimento Andrade Feitosa A, et al. Association between hypertension and quality of life in pregnancy. Hypertens Pregnancy. 2016;35(3):306-14. doi: 10.3109/10641955.2016.1143485
24. Van der Hoorn ML, Lashley EELO, Bianchi DW, et al. Clinical and immunologic aspects of egg donation pregnancies: a systematic review. Human Reproduction Update. 2010;16(6):704-12. doi: 10.1093/humupd/dmq017
25. Harrington K, Carpenter RG, Goldfrad C, et al. Transvaginal Doppler ultrasound of the uteroplacental circulation in the early prediction of pre-eclampsia and intrauterine growth retardation. Br J Obstet Gynaecol. 1997;104(6):674-81. doi: 10.1111/j.1471-0528.1997.tb11977.x
26. Snell KIE, Allotey J, Smuk M, et al. External validation of prognostic models predicting pre-eclampsia: individual participant data meta-analysis. BMC Med. 2020;18(1):302. doi: 10.1186/s12916-020-01766-9
27. Antwi E, Amoakoh-Coleman M, Vieira DL, et al. Systematic review of prediction models for gestational hypertension and preeclampsia. PLoS One. 2020;15(4):e0230955. doi: 10.1371/journal.pone.0230955
28. van Bentem K, Bos M, van der Keur C, et al. The development of preeclampsia in oocyte donation pregnancies is related to the number of fetal-maternal HLA class II mismatches. J Reprod Immunol. 2020;137:103074. doi: 10.1016/j.jri.2019.103074.
29. Lashley EELO, Haasnoot GW, Spruyt-Gerritse M, et al. Selective advantage of HLA matching in successful uncomplicated oocyte donation pregnancies. J Reprod Immunol. 2015;112:29-33. doi: 10.1016/j.jri.2015.05.006

For peer review only - http://bmjopen.bmj.com/site/about/guidelines.xhtml

30. van Bentem K, van der Hoorn ML, van Lith J, et al. Development of hypertensive complications in oocyte donation pregnancy: protocol for a systematic review and individual participant data meta-analysis (DONOR IPD). BMJ Open. 2022;12(7):e059594. doi: 10.1136/bmjopen-2021-059594
31. van Bentem K, Lashley EELO, Bos M, et al. Relating the number of human leucocytes antigen mismatches to pregnancy complications in oocyte donation pregnancies: study protocol for a prospective multicentre cohort study (DONOR study). BMJ Open. 2019;9(7):e027469. doi: 10.1136/bmjopen-2018-027469
32. Castor EDC (Electronic Data Capture). 2023. Available from: http://castoredc.com
33. Dalva B, Meral Beksac. HLA typing with sequence-specific oligonucleotide primed PCR (PCR-SSO)and use of the Luminex technology. Methods Mol Med. 2007;134:61-9. Doi: 10.1007/978-1-4614-9437-9_6
34. Moons KG, Altman DG, Reitsma JB, et al. Transparent Reporting of a multivariable prediction model for Individual Prognosis or Diagnosis (TRIPOD): explanation and elaboration. Ann Intern Med. 2015;162(1):W1-73. doi: 10.7326/m14-0698
35. Collins GS, Reitsma JB, Altman DG, et al. Transparent reporting of a multivariable prediction model for individual prognosis or diagnosis (TRIPOD): the TRIPOD Statement. BMC Medicine. 2015;13(1):1. doi: 10.1186/s12916-014-0241-z
36. Moons KGM, Wolff RF, Riley RD, et al. PROBAST: A Tool to Assess Risk of Bias and Applicability of Prediction Model Studies: Explanation and Elaboration. Ann Intern Med. 2019;170(1):W1-w33. doi: 10.7326/m18-1377
37. van Bentem K, Bos M, van der Keur C, et al. Different immunoregulatory components at the decidua basalis of oocyte donation pregnancies. Hum Immunol. 2022;83(4):319-27. doi: 10.1016/j.humimm.2021.10.008
38. van Diepen M, Ramspek CL, Jager KJ, et al. Prediction versus aetiology: common pitfalls and how to avoid them. Nephrol Dial Transplant. 2017;32(suppl_2):ii1-ii5. doi: 10.1093/ndt/gfw459
39. Steyerberg EW. Clinical Prediction Models: A Practical Approach to Development, Validation, and Updating. New York, NY, USA: Springer; 2009.
40. Riley RD, Ensor J, Snell KIE, et al. Calculating the sample size required for developing a clinical prediction model. BMJ. 2020;368:m441. doi: 10.1136/bmj.m441
41. Riley RD, Snell KIE, Ensor J, et al. Minimum sample size for developing a multivariable prediction model: PART II - binary and time-to-event outcomes. Stat Med. 2019;38(7):1276-96. doi: 10.1002/sim.7992
42. van Smeden M, Moons KG, de Groot JA, et al. Sample size for binary logistic prediction models: Beyond events per variable criteria. Stat Methods Med Res. 2019;28(8):2455-74. doi: 10.1177/0962280218784726
43. Ministry of Health Welfare and Sport (Netherlands). Annual Report 2020 of the Foundation Donor Data Artificial Fertilization. 2020:23.
44. Salama M, Isachenko V, Isachenko E, et al. Cross border reproductive care (CBRC): a growing global phenomenon with multidimensional implications (a systematic and critical review). J Assist Reprod Genet. 2018;35(7):1277-88. doi: 10.1007/s10815-018-1181-x
45. Brown MA, Magee LA, Kenny LC, et al. Hypertensive Disorders of Pregnancy: ISSHP Classification, Diagnosis, and Management Recommendations for International Practice. Hypertension. 2018;72(1):24-43. doi: 10.1161/hypertensionaha.117.10803

For peer review only

For peer review only - http://bmjopen.bmj.com/site/about/guidelines.xhtml

46. Tranquilli D, Deker G, Magee L et al. The classification, diagnosisand management of the hypertensive disorders of pregnancy:a revised statement from the ISSHP. Pregnancy Hypertens. 2014;4:97–104. doi: 10.1016/j.preghy.2014.02.001

47. van Bentem K, Lashley EELO, van der Hoorn ML. Significance of specialised preconception counselling in oocyte donation pregnancy with prior history of postpartum eclampsia. BMJ Case Rep. 2020;13(11). doi: 10.1136/bcr-2020-235582

48. The FIGO Textbook of Pregnancy Hypertension. London: The Global Library of Women's Medicine; 2016.

49. Pimentel C, Solene D, Frédérique J, et al. What are the Predictive Factors for Preeclampsia in Oocyte Recipients? J Hum Reprod Sci. 2019;12(4):327-33. doi: 10.4103/jhrs.JHRS_43_19

50. Steyerberg EW. Clinical Prediction Models - A Practical Approach to Development, Validation, and Updating. Switzerland: Springer Nature Switzerland AG; 2019. 558 p.

51. Steyerberg EW, Vergouwe Y. Towards better clinical prediction models: seven steps for development and an ABCD for validation. Eur Heart J. 2014;35(29):1925-31. doi: 10.1093/eurheartj/ehu207

52. Riley RD, Lambert PC, Abo-Zaid G. Meta-analysis of individual participant data: rationale, conduct, and reporting. BMJ. 2010;340:c221. doi: 10.1136/bmj.c221

53. Stewart LA, Parmar MK. Meta-analysis of the literature or of individual patient data: is there a difference? Lancet. 1993;341(8842):418-22. doi: 10.1016/0140-6736(93)93004-k

54. White IR, Royston P, Wood AM. Multiple imputation using chained equations: Issues and guidance for practice. Stat Med. 2011;30(4):377-99. doi: 10.1002/sim.4067

55. van Ginkel JR, Linting M, Rippe RCA, et al. Rebutting Existing Misconceptions About Multiple Imputation as a Method for Handling Missing Data. J Pers Assess. 2020;102(3):297-308. doi: 10.1080/00223891.2018.1530680

For peer review only - http://bmjopen.bmj.com/site/about/guidelines.xhtml

Author's contributions

EL is the coordinating investigator. MLvdH and EL drafted the protocol and then GL and VvdE wrote the protocol in accordance with the co-authors'contributions. SlC complemented on the epidemiological aspects in this protocol and improved methodology. All authors contributed to the writing of this article and gave final approval of the version to be published.

Funding statement

EL has received the ZonMw Clinical Fellows 2021 Grant, with project code 09032212110062.

Competing interests statement

EL has received the ZonMw Clinical Fellows 2021 Grant, with project code 09032212110062. YL has received honoraria for lectures and presentations by Merck© and Ferring©. Moreover, YL is supported by Gideon Richter© to attend the ORBIS program (a 3 year expert-led program).

Patient and public involvement

During the development of the study protocol, Freya was consulted for input and advice. Freya is the Dutch society for patients with fertility problems and many patients who apply to OD are member of the association. Study information will be published on their website, and information on progress and results will be presented to patients during meetings, patient days and webinars.

For peer review only

For peer review only - http://bmjopen.bmj.com/site/about/guidelines.xhtml

550 **Tables**

551

Table 1. Baseline characteristics

Maternal characteristics	General<br>Date of birth, country of origin, ethnicity, socio-economic status, medical history, family history<br>Obstetric<br>Number of previous pregnancies, number of previous miscarriages/induced abortions/ectopic pregnancies or pregnancies of unknown location, indication for OD treatment<br>Laboratory<br>HLA-typing<br>Oocyte donation procedure<br>Age at time of embryo transfer, BMI at time of embryo transfer, smoking/drugs/alcohol use
Paternal characteristics	General<br>Date of birth, country of origin, ethnicity, medical history, family history<br>Sperm retrieval<br>Sperm from partner vs donor, age at time of sperm retrieval, BMI at time of sperm retrieval, smoking/alcohol/drugs use
Oocyte donor characteristics	Age at time of oocyte retrieval, country where oocyte retrieval took place, ethnicity<br>Laboratory[$]<br>HLA-typing
Procedure	Method used for fertilization (IVF/ICSI), transfer date, country where the transfer took place, transfer in natural or artificial cycle, use of medication (recipient), fresh or cryopreserved transfer of embryo, number of transferred embryos, kind of pregnancy (oocyte donation, embryo donation, or surrogate pregnancy)
Pregnancy	Highest systolic- and diastolic blood pressure, proteinuria, diagnosis of PIH/PE/HELLP, use of salicylic acid during pregnancy, addition or change of medication during pregnancy
Delivery and neonatal characteristics	Place of birth (hospital vs home), mode of delivery, medication use during labour, gestational age, singleton or multiple, gender, birthweight, live birth (yes/no)

552 [$]In case the donor is known and wants to participate, one sample of peripheral blood could be
553 obtained in order to perform HLA-typing.

For peer review only

TRIPOD

TRIPOD Checklist: Prediction Model Development

Section/Topic	Item	Checklist Item	Page
**Title and abstract**			
Title	1	Identify the study as developing and/or validating a multivariable prediction model, the target population, and the outcome to be predicted.	1
Abstract	2	Provide a summary of objectives, study design, setting, participants, sample size, predictors, outcome, statistical analysis, results, and conclusions.	2
**Introduction**			
Background and objectives	3a	Explain the medical context (including whether diagnostic or prognostic) and rationale for developing or validating the multivariable prediction model, including references to existing models.	3
	3b	Specify the objectives, including whether the study describes the development or validation of the model or both.	4
**Methods**			
Source of data	4a	Describe the study design or source of data (e.g., randomized trial, cohort, or registry data), separately for the development and validation data sets, if applicable.	5
	4b	Specify the key study dates, including start of accrual; end of accrual; and, if applicable, end of follow-up.	5
Participants	5a	Specify key elements of the study setting (e.g., primary care, secondary care, general population) including number and location of centres.	5
	5b	Describe eligibility criteria for participants.	5
	5c	Give details of treatments received, if relevant.	n.a.
Outcome	6a	Clearly define the outcome that is predicted by the prediction model, including how and when assessed.	7
	6b	Report any actions to blind assessment of the outcome to be predicted.	n.a.
Predictors	7a	Clearly define all predictors used in developing or validating the multivariable prediction model, including how and when they were measured.	7-8
	7b	Report any actions to blind assessment of predictors for the outcome and other predictors.	n.a.
Sample size	8	Explain how the study size was arrived at.	7
Missing data	9	Describe how missing data were handled (e.g., complete-case analysis, single imputation, multiple imputation) with details of any imputation method.	8
Statistical analysis methods	10a	Describe how predictors were handled in the analyses.	n.a.
	10b	Specify type of model, all model-building procedures (including any predictor selection), and method for internal validation.	8
	10d	Specify all measures used to assess model performance and, if relevant, to compare multiple models.	8
Risk groups	11	Provide details on how risk groups were created, if done.	n.a.
**Results**			
Participants	13a	Describe the flow of participants through the study, including the number of participants with and without the outcome and, if applicable, a summary of the follow-up time. A diagram may be helpful.	n.a.
	13b	Describe the characteristics of the participants (basic demographics, clinical features, available predictors), including the number of participants with missing data for predictors and outcome.	n.a.
Model development	14a	Specify the number of participants and outcome events in each analysis.	n.a.
	14b	If done, report the unadjusted association between each candidate predictor and outcome.	n.a.
Model specification	15a	Present the full prediction model to allow predictions for individuals (i.e., all regression coefficients, and model intercept or baseline survival at a given time point).	n.a.
	15b	Explain how to the use the prediction model.	n.a.
Model performance	16	Report performance measures (with CIs) for the prediction model.	n.a.
**Discussion**			
Limitations	18	Discuss any limitations of the study (such as nonrepresentative sample, few events per predictor, missing data).	9
Interpretation	19b	Give an overall interpretation of the results, considering objectives, limitations, and results from similar studies, and other relevant evidence.	n.a.
Implications	20	Discuss the potential clinical use of the model and implications for future research.	9
**Other information**			
Supplementary information	21	Provide information about the availability of supplementary resources, such as study protocol, Web calculator, and data sets.	n.a.
Funding	22	Give the source of funding and the role of the funders for the present study.	17

We recommend using the TRIPOD Checklist in conjunction with the TRIPOD Explanation and Elaboration document.

For peer review only

STROBE Statement—Checklist of items that should be included in reports of ***cohort studies***

	Item No	Recommendation	Page No
**Title and abstract**	1	(*a*) Indicate the study's design with a commonly used term in the title or the abstract	1
		(*b*) Provide in the abstract an informative and balanced summary of what was done and what was found	2
**Introduction**			
Background/rationale	2	Explain the scientific background and rationale for the investigation being reported	3
Objectives	3	State specific objectives, including any prespecified hypotheses	4
**Methods**			
Study design	4	Present key elements of study design early in the paper	5
Setting	5	Describe the setting, locations, and relevant dates, including periods of recruitment, exposure, follow-up, and data collection	5
Participants	6	(*a*) Give the eligibility criteria, and the sources and methods of selection of participants. Describe methods of follow-up	5
		(*b*) For matched studies, give matching criteria and number of exposed and unexposed	n.a.
Variables	7	Clearly define all outcomes, exposures, predictors, potential confounders, and effect modifiers. Give diagnostic criteria, if applicable	7-8
Data sources/ measurement	8*	For each variable of interest, give sources of data and details of methods of assessment (measurement). Describe comparability of assessment methods if there is more than one group	5
Bias	9	Describe any efforts to address potential sources of bias	6
Study size	10	Explain how the study size was arrived at	7
Quantitative variables	11	Explain how quantitative variables were handled in the analyses. If applicable, describe which groupings were chosen and why	n.a.
Statistical methods	12	(*a*) Describe all statistical methods, including those used to control for confounding	7-8
		(*b*) Describe any methods used to examine subgroups and interactions	n.a.
		(*c*) Explain how missing data were addressed	8
		(*d*) If applicable, explain how loss to follow-up was addressed	n.a.
		(*e*) Describe any sensitivity analyses	n.a.
**Results**			
Participants	13*	(a) Report numbers of individuals at each stage of study—eg numbers potentially eligible, examined for eligibility, confirmed eligible, included in the study, completing follow-up, and analysed	n.a.
		(b) Give reasons for non-participation at each stage	n.a.
		(c) Consider use of a flow diagram	n.a.
Descriptive data	14*	(a) Give characteristics of study participants (eg demographic, clinical, social) and information on exposures and potential confounders	n.a.
		(b) Indicate number of participants with missing data for each variable of interest	n.a.
		(c) Summarise follow-up time (eg, average and total amount)	n.a.
Outcome data	15*	Report numbers of outcome events or summary measures over time	n.a.

For peer review only

For peer review only - http://bmjopen.bmj.com/site/about/guidelines.xhtml

Main results	16	(*a*) Give unadjusted estimates and, if applicable, confounder-adjusted estimates and their precision (eg, 95% confidence interval). Make clear which confounders were adjusted for and why they were included	n.a.
		(*b*) Report category boundaries when continuous variables were categorized	n.a.
		(*c*) If relevant, consider translating estimates of relative risk into absolute risk for a meaningful time period	n.a.
Other analyses	17	Report other analyses done—eg analyses of subgroups and interactions, and sensitivity analyses	n.a.
**Discussion**			
Key results	18	Summarise key results with reference to study objectives	n.a.
Limitations	19	Discuss limitations of the study, taking into account sources of potential bias or imprecision. Discuss both direction and magnitude of any potential bias	9
Interpretation	20	Give a cautious overall interpretation of results considering objectives, limitations, multiplicity of analyses, results from similar studies, and other relevant evidence	n.a.
Generalisability	21	Discuss the generalisability (external validity) of the study results	9
**Other information**			
Funding	22	Give the source of funding and the role of the funders for the present study and, if applicable, for the original study on which the present article is based	17

*Give information separately for exposed and unexposed groups.

Note: An Explanation and Elaboration article discusses each checklist item and gives methodological background and published examples of transparent reporting. The STROBE checklist is best used in conjunction with this article (freely available on the Web sites of PLoS Medicine at http://www.plosmedicine.org/, Annals of Internal Medicine at http://www.annals.org/, and Epidemiology at http://www.epidem.com/). Information on the STROBE Initiative is available at http://www.strobe-statement.org.

For peer review only

BMJ Open

Development of the DONOR prediction model on the risk of hypertensive complications in oocyte donation pregnancy: study protocol for a multicenter cohort study in The Netherlands

Journal:	*BMJ Open*
Manuscript ID	bmjopen-2023-079394.R1
Article Type:	Protocol
Date Submitted by the Author:	19-Feb-2024
Complete List of Authors:	Lafeber, Géraldine; Leiden University Medical Center, Obstetrics & Gynecology<br>Van der Endt, Vera H.W.; Leiden University Medical Center, Obstetrics & Gynaecology<br>Louwers, Yvonne; Erasmus MC, Obstetrics and Gynecology<br>le Cessie, S; Leids Universitair Medisch Centrum, Epidemiology<br>van der Hoorn, Marie-Louise; Leids Universitair Medisch Centrum, Obstetrics & Gynaecology<br>Lashley, Eileen; Leids Universitair Medisch Centrum, Obstetrics & Gynaecology
**Primary Subject Heading**:	Reproductive medicine
Secondary Subject Heading:	Epidemiology, Immunology (including allergy)
Keywords:	Reproductive medicine < GYNAECOLOGY, Hypertension < CARDIOLOGY, Prognosis, Maternal medicine < OBSTETRICS, EPIDEMIOLOGY

SCHOLARONE™ Manuscripts

Development of the DONOR prediction model on the risk of hypertensive complications in oocyte donation pregnancy: study protocol for a multicenter cohort study in The Netherlands

Géraldine C.M. Lafeber[1*], Vera H. W. van der Endt[1*], Yvonne V Louwers[3], S. le Cessie[2], Marie-Louise P. van der Hoorn[1], Eileen E.L.O. Lashley[1]

[1] Department of Obstetrics and Gynecology, Leiden University Medical Center, Leiden, the Netherlands
[2] Department of Clinical Epidemiology, Leiden University Medical Center, Leiden, the Netherlands
[3] Department of Obstetrics and Gynecology, Erasmus Medical Center, the Netherlands

* Authors contributed equally to this manuscript

Keywords
Reproductive medicine, hypertension, prognosis, maternal medicine, epidemiology

Word count
3697

For peer review only

For peer review only - http://bmjopen.bmj.com/site/about/guidelines.xhtml

Abstract

Introduction

Oocyte donation (OD) pregnancy is accompanied with a high incidence of hypertensive complications, with serious consequences for mother and child. Optimal care management, involving early recognition, optimization of suitable treatment options, and possibly eventually also prevention, is of high demand. Prediction of patient specific risk factors for hypertensive complications in OD can provide the basis for this. The current project aims to establish the first prediction model on the risk of hypertensive complications in OD pregnancy.

Methods and analysis

The present study is conducted within the DONation of Oocytes in Reproduction (DONOR) project. For this multicenter cohort study, at least 541 OD pregnancies will be recruited. Baseline characteristics and obstetric data will be collected. Additionally, one sample of maternal peripheral blood and umbilical cord blood after delivery or a saliva sample from the child will be obtained, in order to determine the number of fetal-maternal HLA-mismatches. Following data collection, a multivariate logistic regression model will be developed for the binary outcome hypertensive complication 'yes' and 'no'. The Prediction model Risk Of Bias ASsessment Tool (PROBAST) will be used as guide to minimize the risk of bias. The study will be reported in line with the 'Transparent Reporting of a multivariable prediction model for Individual Prognosis Or Diagnosis (TRIPOD)' guideline. Discrimination and calibration will be determined to assess model performance. Internal validation will be performed using the bootstrapping method. External validation will be performed with the 'DONation of Oocytes in Reproduction individual participant data' (DONOR IPD) dataset.

Ethics and dissemination

This study is approved by the Medical Ethics Committee LDD (Leiden, Den Haag, Delft), with protocol number P16.048 and general assessment registration (ABR) number NL56308.058.16. Further results will be shared through peer-reviewed journals and international conferences

Article summary

Strengths and limitations of this study:

- TRIPOD and PROBAST guidelines will be adhered to, ensuring valid methodology and reporting.
- External validation will be performed using available individual participant data from the DONOR IPD dataset, which will optimize performance evaluation and improve predictive accuracy.
- Assessment of secondary endpoints time of onset and severity of hypertensive complications require larger sample sizes and will therefore be assessed in a later stage.
- The inclusion of a retrospective cohort might introduce bias due to the reliance on medical record, recall bias and incomplete data.
- While 'fetal-maternal HLA (mis)matches' cannot be used as candidate predictor as non-invasive fetal HLA-typing is not yet possible, the individual prognostic value will be assessed.

For peer review only - http://bmjopen.bmj.com/site/about/guidelines.xhtml

Introduction

Subfertility causes a significant socioeconomic burden on global scale. Women facing challenges in generating healthy oocytes for successful pregnancy are posed the opportunity to conceive through oocyte donation (OD)[1]. In OD a donor oocyte is used as opposed to an oocyte retrieved from the intended mother[2, 3]. Since its introduction in 1984[1], ten thousands of OD procedures have been performed per year worldwide, with high success rates[4-8]. It is estimated that over 8% of all IVF cycles are achieved using donated oocytes in Europe[7]. Numbers of OD are rising due to a delay in childbirth which leads to increased maternal age and consequently elevated rates of reproductive issues such as ovarian failure. Further, the expansion of indications for OD use, such as reception of oocytes from partner (ROPA) contribute to the rise of OD numbers [9].

Despite the promising pregnancy and birth rates, however, OD is associated with serious increased obstetric risks. OD pregnancies show a higher incidence of hypertensive complications, such as pregnancy induced hypertension (PIH), preeclampsia (PE) and hemolysis, elevated liver enzymes and low platelets (HELLP) syndrome and other complications due to placental pathology, compared to naturally conceived and IVF pregnancies [8, 10-15]. The chance of developing hypertensive complications in OD pregnancy is more than doubled when compared to conventional IVF and intracytoplasmic sperm injection (ICSI) and at least tripled when compared to naturally conceived pregnancies[10, 14, 16-19]. When translated into absolute risks, the probability of developing hypertensive complications during OD pregnancy ranges from 17% to 18.2% for PIH[14, 18] and from 10.0% to 18% for PE[16, 18, 19].

Although the present-day outcome of hypertensive complications in pregnancy is generally good, PE remains one of the leading causes of maternal death when left untreated [20]. Moreover, hypertensive complications in pregnancy are associated with increased risk of cardiovascular disease and decreased maternal quality of life on the long term [21-23]. Additionally, severe PE poses increased risk for fetal growth restriction and preterm birth, as a result of placental insufficiency [8, 10, 24].

The ability to predict the risk of hypertensive pregnancy complications pre-conceptionally, could potentially aid in disease management and facilitate pre-conceptional counseling. Further, this contributes to limiting unnecessary exposure of the donor to the risks associated with oocyte retrieval. Subsequently, insight in the risk of developing hypertensive disease during pregnancy might improve early recognition and increase insight in the pathophysiology. This could in turn lead to new prevention- and treatment strategies.

Currently, over 100 multivariate prediction models for PE have been developed[25-27], though none of the models is developed nor externally validated for OD pregnancies specifically. As in OD pregnancies a completely allogeneic fetus needs to be tolerated by the mother, these pregnancies differ in essence from natural pregnancy and current predictors might not be applicable in this population. Hence, new predictors such as the genetic differences between mother and child or oocyte donor characteristics, could attribute to the prediction of hypertensive complications in OD pregnancies[11, 28, 29]. As a result, current prediction models might underperform in women conceived through OD, counter-acting the optimalisation of disease management which is strongly needed in this population group. Therefore, we aim to develop and externally validate a multivariate prediction model on the risk of developing hypertensive complications in women considering OD.

For peer review only - http://bmjopen.bmj.com/site/about/guidelines.xhtml

Study objectives

Primary objective

The primary objective of the study is to develop a multivariate prediction model for the development of hypertensive complications in OD pregnancy specifically.

Secondary objectives

Secondary objective is to assess the prognostic effect of individual factors, such as parity, maternal age, fetal-maternal HLA-mismatches, and donor characteristics such as ethnicity and HLA-phenotype, on development of hypertensive complications during pregnancy in women considering OD.

We aim to examine the predictive performances (discrimination, calibration) of our developed multivariate prediction model and to externally validate the model using individual participant data (IPD)[30].

And finally, we aim to explore if it is feasible to develop prediction models for the development of severe PE(including HELLP and early-onset preeclampsia) and time to development of preeclampsia in women considering OD. For this, separate models will be developed not further detailed in this paper.

For peer review only

For peer review only - http://bmjopen.bmj.com/site/about/guidelines.xhtml

Methods and Analysis

Study setting and design

The present study is performed within the DONOR project, on the DONation of Oocytes in Reproduction. This is a multicenter cohort study designed to determine the association between fetal-maternal HLA-mismatches and the development of hypertensive complications in pregnancy [31]. The study will be performed in 7 fertility centers in The Netherlands, with the Leiden University Medical Center as coordinating center.

Eligibility criteria

Patients with the following criteria are eligible for inclusion:

1) Pregnant or delivered after oocyte donation, embryo donation or surrogacy pregnancy. The fertility procedure can be performed in the Netherlands or abroad.
2) Pregnancy duration ≥ 20 weeks.
3) Visit(ed) one of the participating centers.

Patients who are mentally or legally incapable of signing the informed consent form and patients with known chromosomal abnormalities (such as Turner syndrome) or fetal abnormalities will be excluded.

Study population and recruitment

In the prospective cohort, patients who visit the gynecology department of the participating centers will be assessed for eligibility by the attending physician or nurse. Prospective patient recruitment for the DONOR study started at the coordinating center on 1 September 2016. Recruitment at Erasmus MC (EMC) has started in 2019. All other centers will start recruitment in November 2023. Prospective patient recruitment is expected to end on 1 January 2026. In the retrospective cohort, patients who underwent successful OD at one of the participating centers between 2004 and the start of the current study will be approached for participation.

Prior to inclusion, all patients will receive written information and an informed consent form, which comprises a request to obtain permission for gathering data from medical records and a sample of maternal peripheral blood and one sample of umbilical cord blood in the prospective cohort and one sample of saliva of the child in the retrospective cohort. Participants are explicitly informed that participation is entirely voluntary and that they retain the right to withdraw at any time without consequences for subsequent care.

In order to optimize inclusion rate and enabling representation of the Dutch population that applies to OD, more centers will be approached for collaboration.

Data collection

For the prospective cohort, maternal, paternal and donor baseline characteristics and obstetric history will be collected at the initial visit. After delivery, obstetric data will be acquired from the patients' medical records. In the DONOR study, the follow-up period extends from the initial visit until six weeks after delivery. Throughout this period, patients will have regular prenatal and postnatal check-ups in the hospital or primary care, which will be documented in medical records by the attending physician or midwife. Only data from participants with a pregnancy duration ≥20 weeks, will be used in further analysis.

For peer review only - http://bmjopen.bmj.com/site/about/guidelines.xhtml

From the retrospective cohort, maternal baseline and pregnancy characteristics will be collected from the patients' medical records. All available data will be entered into the data management system Castor[32]. An overview of patient data that will be collected is shown in **Table 1**.

Blood and saliva sampling

In the prospective cohort, peripheral maternal blood and umbilical cord blood will be collected for DNA isolation and HLA typing. In the retrospective cohort, peripheral maternal blood and a saliva sample from the child will be obtained. From these samples, DNA will be extracted and HLA-typing will be performed for loci HLA-A, HLA-B, HLA-C, HLA-DQ and HLA-DR, using Reverse Sequence Specific Oligonucleotides PCR technique [33]. Additionally, the number of fetal-maternal HLA-mismatches will be calculated at the national reference laboratory for histocompatibility testing (Leiden University Medical Center, LUMC), based on discrepancy on the HLA-A, -B, -C, -DR and -DQ. The definition of high number of HLA-mismatches is defined as >5 fetal-maternal HLA-mismatches. In case the donor is known and willing to participate, one sample of peripheral blood will be obtained in order to perform HLA-typing as well.

Control of bias

The Prediction model Risk Of Bias Assessment Tool (PROBAST) will be used to minimize the risk of bias [34]. The PROBAST is developed as a general tool for critical appraisal of prediction model studies and consists of 4 domains (participants, predictors, outcome and analysis) containing 20 signaling questions to minimalize risk of bias. In this study most (18/20) PROBAST criteria will be met. The following two PROBAST criteria need attention to minimize the risk of bias.

- Item 1.2 (Domain 1: participants). It is possible that some participants appear multiple times in our dataset, as some participants will conceive through OD more than once. As repeated measurements of the same participant are not independent, robust standard errors will be calculated to account for this. Additionally, using information from medical records poses a risk of information bias and the retrospective cohort could be prone to response and recall bias, for which caution will be taken. Furthermore, we aim to implement the prediction model as a tool for pre-conceptional counseling in women considering OD, whereas development and validation will be performed on a population that is already ≥20 weeks pregnant through OD. We anticipate that this approach will not significantly impact the performance of the prediction model since the majority of covariates remains constant during pregnancy and we expect the time interval between pre-conceptional counseling and pregnancy to be short.
- Item 2.3 (Domain 2: predictors). The predictive value of fetal-maternal HLA-(mis)matches will be assessed, as previous work from our group [28, 29, 35] suggests this could be of significance. However, HLA-typing of the child cannot be performed before or during pregnancy. As the prediction model is intended to be applied in this exact period, the variable of fetal-maternal mismatches will not be used as a candidate predictor in the model. Instead, the variable will be assessed to determine its individual prognostic value on the risk of developing hypertensive disease. Moreover, proximate predictors such as 'the expected amount of fetal-maternal mismatches' based upon the donor HLA typing or 'relation between patient and oocyte donor (familial 'yes' or 'no')' will be used as candidate predictors in our prediction model.

Furthermore, the current study will be reported on in line with the Transparent Reporting of a multivariable prediction model for Individual Prognosis Or Diagnosis (TRIPOD) guideline, which aims

For peer review only - http://bmjopen.bmj.com/site/about/guidelines.xhtml

to improve the reporting of studies developing, validating, or updating a prediction model[36, 37]. Transparent reporting could help mitigate bias.

Sample size calculation

The required sample size was calculated according to the approach of Van Smeden et al. (2019) and Riley et al. (2019, 2020) [38-40]. Based on the current absolute risk of hypertensive complications in patients undergoing OD between 10-20%[14, 16, 18, 19], a number of six candidate predictor parameters, an anticipated model performance of 0.15, a shrinkage factor of 0.90, and a mean absolute prediction error of 0.05 between observed and true outcome probabilities, the required sample size should be at least 541 OD pregnancies.

According to the latest data, approximately 80 successful oocyte or embryo donation procedures are performed yearly in the Netherlands[41]. In addition, a significant number of Dutch women undergo oocyte or embryo donation procedures in countries outside The Netherlands such as Spain, where the laws and regulations concerning these procedures differ[42]. For pregnancy check-ups and delivery, these women return to the Netherlands, which poses the opportunity of participation in our study. Currently, the DONOR cohort [31] consists of 300 OD pregnancies, of which data on fetal-maternal HLA-matching is available for 150 pregnancies. To increase inclusion rate and to optimize representation of the Dutch population that applies to OD, also other centers that perform OD are approached for collaboration. The objective is to collect data from approximately 150 additional pregnancies. Assuming that 50% of all eligible patients visiting one of the participating centers will be included in this study, and that approximately 15% is lost to follow-up or does not reach pregnancy > 20 weeks, it is estimated that the required sample size will be reached within 24 months.

Study outcomes

The primary outcome measure, hypertensive complication in pregnancy, is defined according to the ISSHP classification [43, 44]. PIH is defined as new onset hypertension with diastolic blood pressure ≥90 mmHg and/or systolic blood pressure ≥140 mmHg detected after 20 weeks of gestation. PE is defined as PIH accompanied by one or more of the following: 1) proteinuria, 2) maternal organ dysfunction, 3) uteroplacental dysfunction (fetal growth restriction or abnormal doppler findings)[22]. Severe PE is defined as PE with blood pressure ≥160 mmHg systolic or ≥110 mmHg diastolic, in the presence of HELLP syndrome or in early-onset[44]. HELLP syndrome is defined as hemolysis with a microangiopathic blood smear in combination with elevated liver enzymes and a low platelet count[43]. Early-onset PE is defined as PE occurring before 34 weeks of gestation[44].

To enhance generalizability and applicability of a final model, predictor selection should be well defined, standardized and reproducible. In addition, selection of predictors should be based on literature and clinical reasoning. Therefore the following candidate predictors will be considered[28, 45-47]:

1) Fetal-maternal genetical difference, including
    - Fetal-maternal HLA-(mis)matches (continuous)
    - Relation (family vs non-family) between patient and oocyte donor (dichotomous)
2) Age recipient (continuous)
3) Nulliparity recipient (dichotomous)

For peer review only - http://bmjopen.bmj.com/site/about/guidelines.xhtml

4) Plurality recipient (categorical)
5) BMI recipient (continuous)
6) Smoking recipient (dichotomous)
7) Natural vs artificial cycle (dichotomous)
8) Ethnicity recipient (categorical)
9) Medical history of recipient related to hypertensive complications in pregnancy (dichotomous)
10) Family history of recipient related to hypertensive complications in pregnancy (dichotomous)
11) Age oocyte donor (continuous)
12) Sperm origin (father vs donor) (dichotomous)

Data analysis

All statistical analyses will be performed using R.

Model development

A multivariate logistic regression model will be developed for the binary outcome hypertensive complications in pregnancy 'yes' and 'no'. The model will include the preselected candidate predictors.

As part of the secondary objectives, we aim to predict the risk on developing severe PE and time to development of PE. To achieve this, a multivariate logistic regression model will be developed for the binary outcome severe PE 'yes' or 'no'. Severe PE is defined as blood pressure ≥160 mmHg systolic or ≥110 mmHg diastolic, the presence of HELLP (hemolysis, elevated liver enzymes and low platelets) syndrome or onset of PE before 34 weeks of gestation)[43, 44]. Additionally, in order to predict the time of development of PE, a multivariate linear regression model will be developed for the continuous outcome gestation age at development of PE, with minimum of 20 weeks and maximum of 42 weeks)'. Possible non-linear associations between the continuous predictors and the outcome will be examined, using spline models. Potential multiplicative interaction will be explored by adding interaction terms to the model, and use Lasso regression for variable selection will be considered[48].

Model performance

Internal validation will be performed to assess model performance, discrimination and calibration will be determined. Discrimination describes the ability of the model to discriminate between events and non-events and will be evaluated with the area under the receiver curve (AUC). Calibration describes the relation between the observed risks within the population and the predicted risks and will be assessed by calibration plots, plotting the relation between the observed and predicted risks[49]. Internal validation will be performed using the bootstrapping method with 100 bootstrap samples. The calibration slope from the bootstrapping procedure will be used as uniform shrinkage factor to calculate new regression coefficients to prevent overfitting[48]. External validation will be performed using the 'DONation of Oocytes in Reproduction individual participant data' (DONOR IPD). The DONOR IPD database is currently being set up and will include at least 2301 women pregnant after OD and beyond 20 weeks of gestation[30]. In this study original data from multiple studies will be combined into a single database is therefore suitable for model performance assessment and external validations[50, 51].

Handling of missing data

For peer review only - http://bmjopen.bmj.com/site/about/guidelines.xhtml

In case the amount of missing data in some of the predictors is substantial and cannot be ignored, a multiple imputation method will be applied[52, 53].

Patient and public involvement

During the development of the study protocol, Freya was consulted for input and advice. Freya is the Dutch society for patients with fertility problems and many patients who apply to OD are member of the association. Study information will be published on their website, and information on progress and results will be presented to patients during meetings, patient days and webinars.

For peer review only

For peer review only - http://bmjopen.bmj.com/site/about/guidelines.xhtml

Discussion

Each year, ten thousands of OD procedures are performed successfully worldwide [4-7]. Although the numbers are rising due to delay of maternal age and increasing indications for OD, the method is accompanied with a high incidence of complications, in particular hypertensive disorders of pregnancy. The possibility to predict these complications could potentially aid in disease management and even prevention, yet current prognostic factors and prediction models are not developed, nor validated, for the OD population. With this project, we propose to develop the first prediction model on the risk of hypertensive complications in pregnancy in women considering OD. In clinical practice clinicians could use this developed prediction model to more accurately classify the woman as low or high risk for hypertensive complications in pregnancy, allowing for more personalized counseling for women considering OD. As a consequence this might facilitate in decision making for the patient. In the case the woman is already pregnant, knowledge on hypertensive complications risk might aid the clinician in early recognition of the disease and risk stratification during pregnancy, resulting in better health care management.

One fundamental strength of this project is the adherence to the TRIPOD and PROBAST guidelines, ensuring that essential steps in prediction model development and validation will be performed as recommended [34, 36]. Besides, available IPD [30] makes external validation possible. This will enable optimal performance evaluation and improve predictive accuracy, as populations independent of that used to develop the model will be used with different environmental, geographical, socioeconomic characteristics and different times of conception [30, 37].

It should be acknowledged that control of bias may be impeded as most data will be acquired from medical records. The prediction model will be developed and validated in a population that is already pregnant after OD, whereas the model is intended for facilitating pre-conceptional counseling. Although differences are expected to be small and insignificant, the prediction model might over- or underperform in the intended population group. Therefore, external validation in a population of women considering OD is warranted.

Furthermore, the present project also includes secondary objectives of predicting the severity of PE and its time of onset. The risk of developing severe PE is lower than the risk of hypertensive complication in OD. Additionally, the prediction of time onset of preeclampsia is only possible in patients that actually developed preeclampsia. Larger sample sizes are thus required for these objectives. Accordingly, development of these models will be undertaken during a subsequent phase.

Lastly, the predictor 'fetal-maternal HLA-(mis)matches' cannot be used as a candidate predictor in the model as it is unknown at time of proposed use of the model. The variable will be assessed to determine its individual prognostic value on the risk of developing hypertensive disease. In addition, the expected number of fetal-maternal mismatches could be calculated based on donor and paternal HLA-profiles. Still, the DONOR IPD dataset does not yet contain data on HLA-genotyping, thus the predictive value of fetal-maternal HLA-(mis)matches cannot be validated externally. To enable external validation in future, development of a database including information on fetal and maternal HLA-genotypes is required. We are currently working on a non-invasive method to perform antenatal HLA typing and calculation of mismatches. This will facilitate including the variable in future models and improve the accuracy of our predictions. Additionally, future studies should carefully consider the financial implications of HLA typing as this may impact the feasibility of large-scale implementation and clinical practice. To conclude, this study aims to develop and externally

For peer review only - http://bmjopen.bmj.com/site/about/guidelines.xhtml

validate the first prediction model on the risk of hypertensive complications in pregnancy in women considering OD. The possibility to predict the risk of hypertensive complications in pregnancy could potentially aid in disease management of women considering OD or in women pregnant after OD.

For peer review only

For peer review only - http://bmjopen.bmj.com/site/about/guidelines.xhtml

Ethics and Dissemination

The study will be conducted according to the principles of the Declaration of Helsinki. All patients will obtain written information of the study and in case of participation, the informed consent form should be signed prior to inclusion. This study is approved by the Medical Ethical Committee LDD (Leiden, Den Haag, Delft), with protocol number P16.048 and general assessment registration (ABR) number NL56308.058.16.. All clinical data and biomaterial is pseudonymised by assigning a unique code and will be collected in the data management system Castor. Only a minimum number of members of our project group will have access to this database. The results of our study will be disseminated via publications in gynecology related journals. Further, the results of our study might be presented at (international) conferences.

Ethics statements

Patient consent for publication: not applicable.

For peer review only

For peer review only - http://bmjopen.bmj.com/site/about/guidelines.xhtml

Abbreviations

AUC	Area Under the Curve
DONOR	DONation of Oocytes in Reproduction
HELLP	Heymolysis, Elevated Liver Enzymes and Low Platelets
HLA	Human Leukocyte Antigen
ICSI	Intracytoplasmic Sperm Injection
IPD	Individual Participant Data
IVF	In Vitro Fertilization
LUMC	Leiden University Medical Center
METC	Medical Ethics Committee
NC	Naturally conceived
PE	Preeclampsia
PROBAST	Prediction model Risk Of Bias Assessment Tool
ROB	Risk of Bias
TRIPOD	Transparent Reporting of a multivariable prediction model for Individual Prognosis Or Diagnosis

For peer review only

For peer review only - http://bmjopen.bmj.com/site/about/guidelines.xhtml

References

1. Lutjen P, Trounson A, Leeton J, et al. The establishment and maintenance of pregnancy using in vitro fertilization and embryo donation in a patient with primary ovarian failure. Nature. 1984;307(5947):174-5. doi: 10.1038/307174a0
2. Nederlandse Vereniging voor Klinische Embryologie (KLEM). Modelreglement Embryowet [online]. 2018. https://www.nvog.nl/wp-content/uploads/2020/10/Modelreglement-Embryowet-NVOG-en-KLEM-definitief-augustus-2018-aanpassing-15-oktober-2020.pdf
3. Nederlandse Vereniging voor Obstetrie en Gynaecologie (NVOG). STANDPUNT - Geassisteerde voortplanting met gedoneerde gameten en gedoneerde embryo's en draagmoederschap [online]. 2016. https://www.nvog.nl/wp-content/uploads/2020/06/Standpunt-Geassisteerde-voortplanting-met-gedoneerde-gameten-en-gedoneerde-embryo-juni-2016-addendum-12-6-2020.pdf.
4. Society For Assisted Reproductive Technology. National Summary Report [online]. 2018. https://www.sartcorsonline.com/rptCSR_PublicMultYear.aspx?reportingYear=2018.
5. Hogan RG, Wang AY, Li Z, et al. Oocyte donor age has a significant impact on oocyte recipients' cumulative live-birth rate: a population-based cohort study. Fertil Steril. 2019;112(4):724-30. doi: 10.1016/j.fertnstert.2019.05.012
6. Remohí J, Gartner B, Gallardo E, et al. Pregnancy and birth rates after oocyte donation. Fertil Steril. 1997;67(4):717-23. doi: 10.1016/s0015-0282(97)81372-6
7. Wyns C, De Geyter C, Calhaz-Jorge C, et al. ART in Europe, 2018: results generated from European registries by ESHRE†. Human Reproduction Open. 2022;2022(3). doi: 10.1093/hropen/hoac022
8. Savasi VM, Mandia L, Laoreti A, et al. Maternal and fetal outcomes in oocyte donation pregnancies. Hum Reprod Update. 2016;22(5):620-33. doi: 10.1093/humupd/dmw012
9. Bodri D, Nair S, Gill A et al. Shared motherhood IVF: high quality rates in a large study of treatments for lesbian couples using partner donated eggs. Reprod Biomed Online. 2018;36:130-6. doi: 10.1016/j.rbmo.2017.11.006
10. Storgaard M, Loft A, Bergh C, et al. Obstetric and neonatal complications in pregnancies conceived after oocyte donation: a systematic review and meta-analysis. Bjog. 2017;124(4):561-72. doi: 10.1111/1471-0528.14257
11. Van der Hoorn MP, van Egmond A, Swings G, et al. Differential immunoregulation in successful oocyte donation pregnancies compared with naturally conceived pregnancies. J Reprod Immunol. 2014;101-102:96-103. doi: 10.1016/j.jri.2013.08.002
12. Berntsen S, Larsen EC, la Cour Freiesleben N, et al. Pregnancy outcomes following oocyte donation. Best Pract Res Clin Obstet Gynaecol. 2021;70:81-91. doi: 10.1016/j.bpobgyn.2020.07.008
13. Berntsen S, Söderström-Anttila V, Wennerholm UB, et al. The health of children conceived by ART: 'the chicken or the egg?'. Hum Reprod Update. 2019;25(2):137-58. doi: 10.1093/humupd/dmz001
14. Masoudian P, Nasr A, de Nanassy J, et al. Oocyte donation pregnancies and the risk of preeclampsia or gestational hypertension: a systematic review and metaanalysis. Am J Obstet Gynecol. 2016;214(3):328-39. doi: 10.1016/j.ajog.2015.11.020

For peer review only

15. Levron Y, Dviri M, Segol I, et al. The 'immunologic theory' of preeclampsia revisited: a lesson from donor oocyte gestations. Am J Obstet Gynecol. 2014;211(4):383.e1-5. doi: 10.1016/j.ajog.2014.03.044

16. Blázquez A, García D, Rodríguez A, et al. Is oocyte donation a risk factor for preeclampsia? A systematic review and meta-analysis. J Assist Reprod Genet. 2016;33(7):855-63. doi: 10.1007/s10815-016-0701-9.

17. Moreno-Sepulveda J, Checa MA. Risk of adverse perinatal outcomes after oocyte donation: a systematic review and meta-analysis. J Assist Reprod Genet. 2019;36(10):2017-37. doi: 10.1007/s10815-019-01552-4

18. Jeve YB, Potdar N, Opoku A, et al. Donor oocyte conception and pregnancy complications: a systematic review and meta-analysis. Bjog. 2016;123(9):1471-80. doi: 10.1111/1471-0528.13910

19. Keukens A, van Wely M, van der Meulen C, et al. Pre-eclampsia in pregnancies resulting from oocyte donation, natural conception or IVF: a systematic review and meta-analysis. Hum Reprod. 2022;37(3):586-99. doi: 10.1093/humrep/deab267

20. World Health Organization. Maternal mortality: Key facts [online]. 2021. https://www.who.int/news-room/fact-sheets/detail/maternal-mortality

21. National Institute for Health and Care Excellence (UK): Clinical Guidelines. Hypertension in pregnancy: diagnosis and management [online]. 2019. https://www.nice.org.uk/guidance/ng133

22. Mol BWJ, Roberts CT, Thangaratinam S, et al. Pre-eclampsia. Lancet. 2016;387(10022):999-1011. doi: 10.1016/s0140-6736(15)00070-7

23. Patrícia Medeiros Falcão K, Pedrozo Campos Antunes T, do Nascimento Andrade Feitosa A, et al. Association between hypertension and quality of life in pregnancy. Hypertens Pregnancy. 2016;35(3):306-14. doi: 10.3109/10641955.2016.1143485

24. Van der Hoorn ML, Lashley EELO, Bianchi DW, et al. Clinical and immunologic aspects of egg donation pregnancies: a systematic review. Human Reproduction Update. 2010;16(6):704-12. doi: 10.1093/humupd/dmq017

25. Harrington K, Carpenter RG, Goldfrad C, et al. Transvaginal Doppler ultrasound of the uteroplacental circulation in the early prediction of pre-eclampsia and intrauterine growth retardation. Br J Obstet Gynaecol. 1997;104(6):674-81. doi: 10.1111/j.1471-0528.1997.tb11977.x

26. Snell KIE, Allotey J, Smuk M, et al. External validation of prognostic models predicting pre-eclampsia: individual participant data meta-analysis. BMC Med. 2020;18(1):302. doi: 10.1186/s12916-020-01766-9

27. Antwi E, Amoakoh-Coleman M, Vieira DL, et al. Systematic review of prediction models for gestational hypertension and preeclampsia. PLoS One. 2020;15(4):e0230955. doi: 10.1371/journal.pone.0230955

28. van Bentem K, Bos M, van der Keur C, et al. The development of preeclampsia in oocyte donation pregnancies is related to the number of fetal-maternal HLA class II mismatches. J Reprod Immunol. 2020;137:103074. doi: 10.1016/j.jri.2019.103074.

29. Lashley EELO, Haasnoot GW, Spruyt-Gerritse M, et al. Selective advantage of HLA matching in successful uncomplicated oocyte donation pregnancies. J Reprod Immunol. 2015;112:29-33. doi: 10.1016/j.jri.2015.05.006

For peer review only

For peer review only - http://bmjopen.bmj.com/site/about/guidelines.xhtml

30. van Bentem K, van der Hoorn ML, van Lith J, et al. Development of hypertensive complications in oocyte donation pregnancy: protocol for a systematic review and individual participant data meta-analysis (DONOR IPD). BMJ Open. 2022;12(7):e059594. doi: 10.1136/bmjopen-2021-059594
31. van Bentem K, Lashley EELO, Bos M, et al. Relating the number of human leucocytes antigen mismatches to pregnancy complications in oocyte donation pregnancies: study protocol for a prospective multicentre cohort study (DONOR study). BMJ Open. 2019;9(7):e027469. doi: 10.1136/bmjopen-2018-027469
32. Castor EDC (Electronic Data Capture). 2023. Available from: http://castoredc.com
33. Dalva B, Meral Beksac. HLA typing with sequence-specific oligonucleotide primed PCR (PCR-SSO)and use of the Luminex technology. Methods Mol Med. 2007;134:61-9. Doi: 10.1007/978-1-4614-9437-9_6
34. Moons KG, Altman DG, Reitsma JB, et al. Transparent Reporting of a multivariable prediction model for Individual Prognosis or Diagnosis (TRIPOD): explanation and elaboration. Ann Intern Med. 2015;162(1):W1-73. doi: 10.7326/m14-0698
35. van Bentem K, Bos M, van der Keur C, et al. Different immunoregulatory components at the decidua basalis of oocyte donation pregnancies. Hum Immunol. 2022;83(4):319-27. doi: 10.1016/j.humimm.2021.10.008.
36. Collins GS, Reitsma JB, Altman DG, et al. Transparent reporting of a multivariable prediction model for individual prognosis or diagnosis (TRIPOD): the TRIPOD Statement. BMC Medicine. 2015;13(1):1. doi: 10.1186/s12916-014-0241-z
37. Moons KGM, Wolff RF, Riley RD, et al. PROBAST: A Tool to Assess Risk of Bias and Applicability of Prediction Model Studies: Explanation and Elaboration. Ann Intern Med. 2019;170(1):W1-w33. doi: 10.7326/m18-1377
38. Riley RD, Ensor J, Snell KIE, et al. Calculating the sample size required for developing a clinical prediction model. BMJ. 2020;368:m441. doi: 10.1136/bmj.m441
39. Riley RD, Snell KIE, Ensor J, et al. Minimum sample size for developing a multivariable prediction model: PART II - binary and time-to-event outcomes. Stat Med. 2019;38(7):1276-96. doi: 10.1002/sim.7992
40. van Smeden M, Moons KG, de Groot JA, et al. Sample size for binary logistic prediction models: Beyond events per variable criteria. Stat Methods Med Res. 2019;28(8):2455-74. doi: 10.1177/0962280218784726
41. Ministry of Health Welfare and Sport (Netherlands). Annual Report 2020 of the Foundation Donor Data Artificial Fertilization. 2020:23.
42. Salama M, Isachenko V, Isachenko E, et al. Cross border reproductive care (CBRC): a growing global phenomenon with multidimensional implications (a systematic and critical review). J Assist Reprod Genet. 2018;35(7):1277-88. doi: 10.1007/s10815-018-1181-x
43. Brown MA, Magee LA, Kenny LC, et al. Hypertensive Disorders of Pregnancy: ISSHP Classification, Diagnosis, and Management Recommendations for International Practice. Hypertension. 2018;72(1):24-43. doi: 10.1161/hypertensionaha.117.10803
44. Tranquilli D, Deker G, Magee L et al. The classification, diagnosis and management of the hypertensive disorders of pregnancy: a revised statement from the ISSHP. Pregnancy Hypertens. 2014;4:97–104. doi: 10.1016/j.preghy.2014.02.001

For peer review only

For peer review only - http://bmjopen.bmj.com/site/about/guidelines.xhtml

45. van Bentem K, Lashley EELO, van der Hoorn ML. Significance of specialised preconception counselling in oocyte donation pregnancy with prior history of postpartum eclampsia. BMJ Case Rep. 2020;13(11). doi: 10.1136/bcr-2020-235582
46. The FIGO Textbook of Pregnancy Hypertension. London: The Global Library of Women's Medicine; 2016.
47. Pimentel C, Solene D, Frédérique J, et al. What are the Predictive Factors for Preeclampsia in Oocyte Recipients? J Hum Reprod Sci. 2019;12(4):327-33. doi: 10.4103/jhrs.JHRS_43_19
48. Steyerberg EW. Clinical Prediction Models - A Practical Approach to Development, Validation, and Updating. Switzerland: Springer Nature Switzerland AG; 2019. 558 p.
49. Steyerberg EW, Vergouwe Y. Towards better clinical prediction models: seven steps for development and an ABCD for validation. Eur Heart J. 2014;35(29):1925-31. doi: 10.1093/eurheartj/ehu207
50. Riley RD, Lambert PC, Abo-Zaid G. Meta-analysis of individual participant data: rationale, conduct, and reporting. BMJ. 2010;340:c221. doi: 10.1136/bmj.c221
51. Stewart LA, Parmar MK. Meta-analysis of the literature or of individual patient data: is there a difference? Lancet. 1993;341(8842):418-22. doi: 10.1016/0140-6736(93)93004-k
52. White IR, Royston P, Wood AM. Multiple imputation using chained equations: Issues and guidance for practice. Stat Med. 2011;30(4):377-99. doi: 10.1002/sim.4067
53. van Ginkel JR, Linting M, Rippe RCA, et al. Rebutting Existing Misconceptions About Multiple Imputation as a Method for Handling Missing Data. J Pers Assess. 2020;102(3):297-308. doi: 10.1080/00223891.2018.1530680

For peer review only

For peer review only - http://bmjopen.bmj.com/site/about/guidelines.xhtml

Author's contributions

MLvdH and EL drafted the protocol, with SlC making substantial contributions to the epidemiological design of the work. EL and YL are the coordinating investigators in the LUMC and the Erasmus MC respectively. GL and VvdE wrote the protocol in accordance with the co-authors' contributions. GL is the corresponding author and submitted the manuscript. All authors gave final approval of the version to be published and agree to be accountable for all aspects of the work.

Funding statement

EL has received the ZonMw Clinical Fellows 2021 Grant, with project code 09032212110062.

Competing interests statement

EL has received the ZonMw Clinical Fellows 2021 Grant, with project code 09032212110062. YL has received honoraria for lectures and presentations by Merck© and Ferring©. Moreover, YL is supported by Gideon Richter© to attend the ORBIS program (a 3 year expert-led program). GL, VvdE, SlC and MLvdH have no competing interests.

For peer review only

For peer review only - http://bmjopen.bmj.com/site/about/guidelines.xhtml

Tables

Table 1. Baseline characteristics

Maternal characteristics	General<br>Date of birth, country of origin, ethnicity, socio-economic status, medical history, family history<br>Obstetric<br>Number of previous pregnancies, number of previous miscarriages/induced abortions/ectopic pregnancies or pregnancies of unknown location, indication for OD treatment<br>Laboratory<br>HLA-typing<br>Oocyte donation procedure<br>Age at time of embryo transfer, BMI at time of embryo transfer, smoking/drugs/alcohol use
Paternal characteristics	General<br>Date of birth, country of origin, ethnicity, medical history, family history<br>Sperm retrieval<br>Sperm from partner vs donor, age at time of sperm retrieval, BMI at time of sperm retrieval, smoking/alcohol/drugs use
Oocyte donor characteristics	Age at time of oocyte retrieval, country where oocyte retrieval took place, ethnicity<br>Laboratory[$]<br>HLA-typing
Procedure	Method used for fertilization (IVF/ICSI), transfer date, country where the transfer took place, transfer in natural or artificial cycle, use of medication (recipient), fresh or cryopreserved transfer of embryo, number of transferred embryos, kind of pregnancy (oocyte donation, embryo donation, or surrogate pregnancy)
Pregnancy	Highest systolic- and diastolic blood pressure, proteinuria, diagnosis of PIH/PE/HELLP, use of salicylic acid during pregnancy, addition or change of medication during pregnancy
Delivery and neonatal characteristics	Place of birth (hospital vs home), mode of delivery, medication use during labour, gestational age, singleton or multiple, gender, birthweight, live birth (yes/no)

[$]In case the donor is known and wants to participate, one sample of peripheral blood could be obtained in order to perform HLA-typing.

For peer review only

For peer review only - http://bmjopen.bmj.com/site/about/guidelines.xhtml

TRIPOD Checklist: Prediction Model Development

Section/Topic	Item	Checklist Item	Page
**Title and abstract**			
Title	1	Identify the study as developing and/or validating a multivariable prediction model, the target population, and the outcome to be predicted.	1
Abstract	2	Provide a summary of objectives, study design, setting, participants, sample size, predictors, outcome, statistical analysis, results, and conclusions.	2
**Introduction**			
Background and objectives	3a	Explain the medical context (including whether diagnostic or prognostic) and rationale for developing or validating the multivariable prediction model, including references to existing models.	3
	3b	Specify the objectives, including whether the study describes the development or validation of the model or both.	4
**Methods**			
Source of data	4a	Describe the study design or source of data (e.g., randomized trial, cohort, or registry data), separately for the development and validation data sets, if applicable.	5
	4b	Specify the key study dates, including start of accrual; end of accrual; and, if applicable, end of follow-up.	5
Participants	5a	Specify key elements of the study setting (e.g., primary care, secondary care, general population) including number and location of centres.	5
	5b	Describe eligibility criteria for participants.	5
	5c	Give details of treatments received, if relevant.	n.a.
Outcome	6a	Clearly define the outcome that is predicted by the prediction model, including how and when assessed.	7
	6b	Report any actions to blind assessment of the outcome to be predicted.	n.a.
Predictors	7a	Clearly define all predictors used in developing or validating the multivariable prediction model, including how and when they were measured.	7-8
	7b	Report any actions to blind assessment of predictors for the outcome and other predictors.	n.a.
Sample size	8	Explain how the study size was arrived at.	7
Missing data	9	Describe how missing data were handled (e.g., complete-case analysis, single imputation, multiple imputation) with details of any imputation method.	8
Statistical analysis methods	10a	Describe how predictors were handled in the analyses.	n.a.
	10b	Specify type of model, all model-building procedures (including any predictor selection), and method for internal validation.	8
	10d	Specify all measures used to assess model performance and, if relevant, to compare multiple models.	8
Risk groups	11	Provide details on how risk groups were created, if done.	n.a.
**Results**			
Participants	13a	Describe the flow of participants through the study, including the number of participants with and without the outcome and, if applicable, a summary of the follow-up time. A diagram may be helpful.	n.a.
	13b	Describe the characteristics of the participants (basic demographics, clinical features, available predictors), including the number of participants with missing data for predictors and outcome.	n.a.
Model development	14a	Specify the number of participants and outcome events in each analysis.	n.a.
	14b	If done, report the unadjusted association between each candidate predictor and outcome.	n.a.
Model specification	15a	Present the full prediction model to allow predictions for individuals (i.e., all regression coefficients, and model intercept or baseline survival at a given time point).	n.a.
	15b	Explain how to the use the prediction model.	n.a.
Model performance	16	Report performance measures (with CIs) for the prediction model.	n.a.
**Discussion**			
Limitations	18	Discuss any limitations of the study (such as nonrepresentative sample, few events per predictor, missing data).	9
Interpretation	19b	Give an overall interpretation of the results, considering objectives, limitations, and results from similar studies, and other relevant evidence.	n.a.
Implications	20	Discuss the potential clinical use of the model and implications for future research.	9
**Other information**			
Supplementary information	21	Provide information about the availability of supplementary resources, such as study protocol, Web calculator, and data sets.	n.a.
Funding	22	Give the source of funding and the role of the funders for the present study.	17

We recommend using the TRIPOD Checklist in conjunction with the TRIPOD Explanation and Elaboration document.

STROBE Statement—Checklist of items that should be included in reports of ***cohort studies***

	Item No	Recommendation	Page No
**Title and abstract**	1	(*a*) Indicate the study's design with a commonly used term in the title or the abstract	1
		(*b*) Provide in the abstract an informative and balanced summary of what was done and what was found	2
**Introduction**			
Background/rationale	2	Explain the scientific background and rationale for the investigation being reported	3
Objectives	3	State specific objectives, including any prespecified hypotheses	4
**Methods**			
Study design	4	Present key elements of study design early in the paper	5
Setting	5	Describe the setting, locations, and relevant dates, including periods of recruitment, exposure, follow-up, and data collection	5
Participants	6	(*a*) Give the eligibility criteria, and the sources and methods of selection of participants. Describe methods of follow-up	5
		(*b*) For matched studies, give matching criteria and number of exposed and unexposed	n.a.
Variables	7	Clearly define all outcomes, exposures, predictors, potential confounders, and effect modifiers. Give diagnostic criteria, if applicable	7-8
Data sources/ measurement	8*	For each variable of interest, give sources of data and details of methods of assessment (measurement). Describe comparability of assessment methods if there is more than one group	5
Bias	9	Describe any efforts to address potential sources of bias	6
Study size	10	Explain how the study size was arrived at	7
Quantitative variables	11	Explain how quantitative variables were handled in the analyses. If applicable, describe which groupings were chosen and why	n.a.
Statistical methods	12	(*a*) Describe all statistical methods, including those used to control for confounding	7-8
		(*b*) Describe any methods used to examine subgroups and interactions	n.a.
		(*c*) Explain how missing data were addressed	8
		(*d*) If applicable, explain how loss to follow-up was addressed	n.a.
		(*e*) Describe any sensitivity analyses	n.a.
**Results**			
Participants	13*	(a) Report numbers of individuals at each stage of study—eg numbers potentially eligible, examined for eligibility, confirmed eligible, included in the study, completing follow-up, and analysed	n.a.
		(b) Give reasons for non-participation at each stage	n.a.
		(c) Consider use of a flow diagram	n.a.
Descriptive data	14*	(a) Give characteristics of study participants (eg demographic, clinical, social) and information on exposures and potential confounders	n.a.
		(b) Indicate number of participants with missing data for each variable of interest	n.a.
		(c) Summarise follow-up time (eg, average and total amount)	n.a.
Outcome data	15*	Report numbers of outcome events or summary measures over time	n.a.

For peer review only

Main results	16	(*a*) Give unadjusted estimates and, if applicable, confounder-adjusted estimates and their precision (eg, 95% confidence interval). Make clear which confounders were adjusted for and why they were included	n.a.
		(*b*) Report category boundaries when continuous variables were categorized	n.a.
		(*c*) If relevant, consider translating estimates of relative risk into absolute risk for a meaningful time period	n.a.
Other analyses	17	Report other analyses done—eg analyses of subgroups and interactions, and sensitivity analyses	n.a.
**Discussion**			
Key results	18	Summarise key results with reference to study objectives	n.a.
Limitations	19	Discuss limitations of the study, taking into account sources of potential bias or imprecision. Discuss both direction and magnitude of any potential bias	9
Interpretation	20	Give a cautious overall interpretation of results considering objectives, limitations, multiplicity of analyses, results from similar studies, and other relevant evidence	n.a.
Generalisability	21	Discuss the generalisability (external validity) of the study results	9
**Other information**			
Funding	22	Give the source of funding and the role of the funders for the present study and, if applicable, for the original study on which the present article is based	17

*Give information separately for exposed and unexposed groups.

Note: An Explanation and Elaboration article discusses each checklist item and gives methodological background and published examples of transparent reporting. The STROBE checklist is best used in conjunction with this article (freely available on the Web sites of PLoS Medicine at http://www.plosmedicine.org/, Annals of Internal Medicine at http://www.annals.org/, and Epidemiology at http://www.epidem.com/). Information on the STROBE Initiative is available at http://www.strobe-statement.org.

For peer review only

BMJ Open

Development of the DONOR prediction model on the risk of hypertensive complications in oocyte donation pregnancy: study protocol for a multicenter cohort study in The Netherlands

Journal:	*BMJ Open*
Manuscript ID	bmjopen-2023-079394.R2
Article Type:	Protocol
Date Submitted by the Author:	15-Apr-2024

Complete List of Authors:	Lafeber, Géraldine; Leiden University Medical Center, Obstetrics & Gynecology<br>Van der Endt, Vera H.W.; Leiden University Medical Center, Obstetrics & Gynaecology<br>Louwers, Yvonne; Erasmus MC, Obstetrics and Gynecology<br>le Cessie, S; Leids Universitair Medisch Centrum, Epidemiology<br>van der Hoorn, Marie-Louise; Leids Universitair Medisch Centrum, Obstetrics & Gynaecology<br>Lashley, Eileen; Leids Universitair Medisch Centrum, Obstetrics & Gynaecology

**Primary Subject Heading**:	Reproductive medicine
Secondary Subject Heading:	Epidemiology, Immunology (including allergy)
Keywords:	Reproductive medicine < GYNAECOLOGY, Hypertension < CARDIOLOGY, Prognosis, Maternal medicine < OBSTETRICS, EPIDEMIOLOGY

SCHOLARONE™
Manuscripts

Development of the DONOR prediction model on the risk of hypertensive complications in oocyte donation pregnancy: study protocol for a multicenter cohort study in The Netherlands

Géraldine C.M. Lafeber[1*], Vera H. W. van der Endt[1*], Yvonne V Louwers[3], S. le Cessie[2], Marie-Louise P. van der Hoorn[1], Eileen E.L.O. Lashley[1]

[1] Department of Obstetrics and Gynecology, Leiden University Medical Center, Leiden, the Netherlands
[2] Department of Clinical Epidemiology, Leiden University Medical Center, Leiden, the Netherlands
[3] Department of Obstetrics and Gynecology, Erasmus Medical Center, the Netherlands

* Authors contributed equally to this manuscript

Corresponding author: Géraldine C.M. Lafeber, g.c.m.lafeber@lumc.nl

Keywords
Reproductive medicine, hypertension, prognosis, maternal medicine, epidemiology

Word count
3617

For peer review only

Abstract

Introduction

Oocyte donation (OD) pregnancy is accompanied with a high incidence of hypertensive complications, with serious consequences for mother and child. Optimal care management, involving early recognition, optimization of suitable treatment options, and possibly eventually also prevention, is of high demand. Prediction of patient specific risk factors for hypertensive complications in OD can provide the basis for this. The current project aims to establish the first prediction model on the risk of hypertensive complications in OD pregnancy.

Methods and analysis

The present study is conducted within the DONation of Oocytes in Reproduction (DONOR) project. For this multicenter cohort study, at least 541 OD pregnancies will be recruited. Baseline characteristics and obstetric data will be collected. Additionally, one sample of maternal peripheral blood and umbilical cord blood after delivery or a saliva sample from the child will be obtained, in order to determine the number of fetal-maternal HLA-mismatches. Following data collection, a multivariate logistic regression model will be developed for the binary outcome hypertensive complication 'yes' and 'no'. The Prediction model Risk Of Bias ASsessment Tool (PROBAST) will be used as guide to minimize the risk of bias. The study will be reported in line with the 'Transparent Reporting of a multivariable prediction model for Individual Prognosis Or Diagnosis (TRIPOD)' guideline. Discrimination and calibration will be determined to assess model performance. Internal validation will be performed using the bootstrapping method. External validation will be performed with the 'DONation of Oocytes in Reproduction individual participant data' (DONOR IPD) dataset.

Ethics and dissemination

This study is approved by the Medical Ethics Committee LDD (Leiden, Den Haag, Delft), with protocol number P16.048 and general assessment registration (ABR) number NL56308.058.16. Further results will be shared through peer-reviewed journals and international conferences

Article summary

Strengths and limitations of this study:

- TRIPOD and PROBAST guidelines will be adhered to, ensuring valid methodology and reporting.
- External validation will be performed using available individual participant data from the DONOR IPD dataset, which will optimize performance evaluation and improve predictive accuracy.
- Assessment of secondary endpoints time of onset and severity of hypertensive complications require larger sample sizes and will therefore be assessed in a later stage.
- The inclusion of a retrospective cohort might introduce bias due to the reliance on medical record, recall bias and incomplete data.
- While 'fetal-maternal HLA (mis)matches' cannot be used as candidate predictor as non-invasive fetal HLA-typing is not yet possible, the individual prognostic value will be assessed.

For peer review only - http://bmjopen.bmj.com/site/about/guidelines.xhtml

Introduction

Subfertility causes a significant socioeconomic burden on global scale. Women facing challenges in generating healthy oocytes for successful pregnancy are posed the opportunity to conceive through oocyte donation (OD)(1). In OD a donor oocyte is used as opposed to an oocyte retrieved from the intended mother(2, 3). Since its introduction in 1984(1), ten thousands of OD procedures have been performed per year worldwide, with high success rates(4-8). It is estimated that over 8% of all IVF cycles are achieved using donated oocytes in Europe(7). Numbers of OD are rising due to a delay in childbirth which leads to increased maternal age and consequently elevated rates of reproductive issues such as ovarian failure. Further, the expansion of indications for OD use, such as reception of oocytes from partner (ROPA) contribute to the rise of OD numbers (9).

Despite the promising pregnancy and birth rates, however, OD is associated with serious increased obstetric risks. OD pregnancies show a higher incidence of hypertensive complications, such as pregnancy induced hypertension (PIH), preeclampsia (PE) and hemolysis, elevated liver enzymes and low platelets (HELLP) syndrome and other complications due to placental pathology, compared to naturally conceived and IVF pregnancies (8, 10-15). The chance of developing hypertensive complications in OD pregnancy is more than doubled when compared to conventional IVF and intracytoplasmic sperm injection (ICSI) and at least tripled when compared to naturally conceived pregnancies(10, 14, 16-19). When translated into absolute risks, the probability of developing hypertensive complications during OD pregnancy ranges from 17% to 18.2% for PIH(14, 18) and from 10.0% to 18% for PE(16, 18, 19).

Although the present-day outcome of hypertensive complications in pregnancy is generally good, PE remains one of the leading causes of maternal death when left untreated (20). Moreover, hypertensive complications in pregnancy are associated with increased risk of cardiovascular disease and decreased maternal quality of life on the long term (21-23). Additionally, severe PE poses increased risk for fetal growth restriction and preterm birth, as a result of placental insufficiency (8, 10, 24).

The ability to predict the risk of hypertensive pregnancy complications pre-conceptionally, could potentially aid in disease management and facilitate pre-conceptional counseling. Further, this contributes to limiting unnecessary exposure of the donor to the risks associated with oocyte retrieval. Subsequently, insight in the risk of developing hypertensive disease during pregnancy might improve early recognition and increase insight in the pathophysiology. This could in turn lead to new prevention- and treatment strategies.

Currently, over 100 multivariate prediction models for PE have been developed(25-27), though none of the models is developed nor externally validated for OD pregnancies specifically. As in OD pregnancies a completely allogeneic fetus needs to be tolerated by the mother, these pregnancies differ in essence from natural pregnancy and current predictors might not be applicable in this population. Hence, new predictors such as the genetic differences between mother and child or oocyte donor characteristics, could attribute to the prediction of hypertensive complications in OD pregnancies(11, 28, 29). As a result, current prediction models might underperform in women conceived through OD, counter-acting the optimalisation of disease management which is strongly needed in this population group. Therefore, we aim to develop and externally validate a multivariate prediction model on the risk of developing hypertensive complications in women considering OD.

For peer review only - http://bmjopen.bmj.com/site/about/guidelines.xhtml

Study objectives

Primary objective

The primary objective of the study is to develop a multivariate prediction model for the development of hypertensive complications in OD pregnancy specifically.

Secondary objectives

Secondary objective is to assess the prognostic effect of individual factors, such as parity, maternal age, fetal-maternal HLA-mismatches, and donor characteristics such as ethnicity and HLA-phenotype, on development of hypertensive complications during pregnancy in women considering OD.

We aim to examine the predictive performances (discrimination, calibration) of our developed multivariate prediction model and to externally validate the model using individual participant data (IPD)(30).

And finally, we aim to explore if it is feasible to develop prediction models for the development of HELLP and early-onset preeclampsia and time to development of preeclampsia in women considering OD. For this, separate models will be developed not further detailed in this paper.

For peer review only

For peer review only - http://bmjopen.bmj.com/site/about/guidelines.xhtml

Methods and Analysis

Study setting and design

The present study is performed within the DONOR project, on the DONation of Oocytes in Reproduction. This is a multicenter cohort study designed to determine the association between fetal-maternal HLA-mismatches and the development of hypertensive complications in pregnancy (31). The study will be performed in 7 fertility centers in The Netherlands, with the Leiden University Medical Center as coordinating center.

Eligibility criteria

Patients with the following criteria are eligible for inclusion:

1) Pregnant or delivered after oocyte donation, embryo donation or surrogacy pregnancy. The fertility procedure can be performed in the Netherlands or abroad.
2) Pregnancy duration ≥ 20 weeks.
3) Visit(ed) one of the participating centers.

Patients who are mentally or legally incapable of signing the informed consent form and patients with known chromosomal abnormalities (such as Turner syndrome) or fetal abnormalities will be excluded.

Study population and recruitment

In the prospective cohort, patients who visit the gynecology department of the participating centers will be assessed for eligibility by the attending physician or nurse. Prospective patient recruitment for the DONOR study started at the coordinating center on 1 September 2016. Recruitment at Erasmus MC (EMC) has started in 2019. All other centers will start recruitment in November 2023. Prospective patient recruitment is expected to end on 1 January 2026. In the retrospective cohort, patients who underwent successful OD at one of the participating centers between 2004 and the start of the current study will be approached for participation.

Prior to inclusion, all patients will receive written information and an informed consent form, which comprises a request to obtain permission for gathering data from medical records and a sample of maternal peripheral blood and one sample of umbilical cord blood in the prospective cohort and one sample of saliva of the child in the retrospective cohort. Participants are explicitly informed that participation is entirely voluntary and that they retain the right to withdraw at any time without consequences for subsequent care.

In order to optimize inclusion rate and enabling representation of the Dutch population that applies to OD, more centers will be approached for collaboration.

Data collection

For the prospective cohort, maternal, paternal and donor baseline characteristics and obstetric history will be collected at the initial visit. After delivery, obstetric data will be acquired from the patients' medical records. In the DONOR study, the follow-up period extends from the initial visit until six weeks after delivery. Throughout this period, patients will have regular prenatal and postnatal check-ups in the hospital or primary care, which will be documented in medical records by the attending physician or midwife. Only data from participants with a pregnancy duration ≥20 weeks, will be used in further analysis.

For peer review only - http://bmjopen.bmj.com/site/about/guidelines.xhtml

From the retrospective cohort, maternal baseline and pregnancy characteristics will be collected from the patients' medical records. All available data will be entered into the data management system Castor(32). An overview of patient data that will be collected is shown in **Table 1**.

Blood and saliva sampling

In the prospective cohort, peripheral maternal blood and umbilical cord blood will be collected for DNA isolation and HLA typing. In the retrospective cohort, peripheral maternal blood and a saliva sample from the child will be obtained. From these samples, DNA will be extracted and HLA-typing will be performed for loci HLA-A, HLA-B, HLA-C, HLA-DQ and HLA-DR, using Reverse Sequence Specific Oligonucleotides PCR technique (33). Additionally, the number of fetal-maternal HLA-mismatches will be calculated at the national reference laboratory for histocompatibility testing (Leiden University Medical Center, LUMC), based on discrepancy on the HLA-A, -B, -C, -DR and -DQ. The definition of high number of HLA-mismatches is defined as >5 fetal-maternal HLA-mismatches. In case the donor is known and willing to participate, one sample of peripheral blood will be obtained in order to perform HLA-typing as well.

Control of bias

The Prediction model Risk Of Bias Assessment Tool (PROBAST) will be used to minimize the risk of bias (34). The PROBAST is developed as a general tool for critical appraisal of prediction model studies and consists of 4 domains (participants, predictors, outcome and analysis) containing 20 signaling questions to minimalize risk of bias. In this study most (18/20) PROBAST criteria will be met. The following two PROBAST criteria need attention to minimize the risk of bias.

- Item 1.2 (Domain 1: participants). It is possible that some participants appear multiple times in our dataset, as some participants will conceive through OD more than once. As repeated measurements of the same participant are not independent, robust standard errors will be calculated to account for this. Additionally, using information from medical records poses a risk of information bias and the retrospective cohort could be prone to response and recall bias, for which caution will be taken. Furthermore, we aim to implement the prediction model as a tool for pre-conceptional counseling in women considering OD, whereas development and validation will be performed on a population that is already ≥20 weeks pregnant through OD. We anticipate that this approach will not significantly impact the performance of the prediction model since the majority of covariates remains constant during pregnancy and we expect the time interval between pre-conceptional counseling and pregnancy to be short.

- Item 2.3 (Domain 2: predictors). The predictive value of fetal-maternal HLA-(mis)matches will be assessed, as previous work from our group (28, 29, 35) suggests this could be of significance. However, HLA-typing of the child cannot be performed before or during pregnancy. As the prediction model is intended to be applied in this exact period, the variable of fetal-maternal mismatches will not be used as a candidate predictor in the model. Instead, the variable will be assessed to determine its individual prognostic value on the risk of developing hypertensive disease. Moreover, proximate predictors such as 'the expected amount of fetal-maternal mismatches' based upon the donor HLA typing or 'relation between patient and oocyte donor (familial 'yes' or 'no')' will be used as candidate predictors in our prediction model.

Furthermore, the current study will be reported on in line with the Transparent Reporting of a multivariable prediction model for Individual Prognosis Or Diagnosis (TRIPOD) guideline, which aims

For peer review only

For peer review only - http://bmjopen.bmj.com/site/about/guidelines.xhtml

to improve the reporting of studies developing, validating, or updating a prediction model(36, 37). Transparent reporting could help mitigate bias.

Sample size calculation

The required sample size was calculated according to the approach of Van Smeden et al. (2019) and Riley et al. (2019, 2020) (38-40). Based on the current absolute risk of hypertensive complications in patients undergoing OD between 10-20%(14, 16, 18, 19), a number of six candidate predictor parameters, an anticipated model performance of 0.15, a shrinkage factor of 0.90, and a mean absolute prediction error of 0.05 between observed and true outcome probabilities, the required sample size should be at least 541 OD pregnancies.

According to the latest data, approximately 80 successful oocyte or embryo donation procedures are performed yearly in the Netherlands(41). In addition, a significant number of Dutch women undergo oocyte or embryo donation procedures in countries outside The Netherlands such as Spain, where the laws and regulations concerning these procedures differ(42). For pregnancy check-ups and delivery, these women return to the Netherlands, which poses the opportunity of participation in our study. Currently, the DONOR cohort (31) consists of 300 OD pregnancies, of which data on fetal-maternal HLA-matching is available for 150 pregnancies. To increase inclusion rate and to optimize representation of the Dutch population that applies to OD, also other centers that perform OD are approached for collaboration. The objective is to collect data from approximately 150 additional pregnancies. Assuming that 50% of all eligible patients visiting one of the participating centers will be included in this study, and that approximately 15% is lost to follow-up or does not reach pregnancy > 20 weeks, it is estimated that the required sample size will be reached within 24 months.

Study outcomes

The primary outcome measure, hypertensive complication in pregnancy, is defined according to the 2021 ISSHP classification(43). PIH is defined as new onset hypertension with diastolic blood pressure ≥90 mmHg and/or systolic blood pressure ≥140 mmHg detected after 20 weeks of gestation. PE is defined as PIH accompanied by one or more of the following: 1) proteinuria, 2) maternal organ dysfunction, 3) uteroplacental dysfunction (fetal growth restriction or abnormal doppler findings)(22, 43). Superimposed PE is defined by chronic hypertension combined with evidence of uteroplacental dysfunction. Severe hypertension is defined as blood pressure ≥ 160mmHg systolic or ≥110mmHg diastolic. HELLP syndrome, which is a serious manifestation of PE, is defined as hemolysis with a microangiopathic blood smear in combination with elevated liver enzymes and a low platelet count (43).

To enhance generalizability and applicability of a final model, predictor selection should be well defined, standardized and reproducible. In addition, selection of predictors should be based on literature and clinical reasoning. Therefore the following candidate predictors will be considered(28, 44-46):

1) Fetal-maternal genetical difference, including
   - Fetal-maternal HLA-(mis)matches (continuous)
   - Relation (family vs non-family) between patient and oocyte donor (dichotomous)

2) Age recipient (continuous)

For peer review only - http://bmjopen.bmj.com/site/about/guidelines.xhtml

3) Nulliparity recipient (dichotomous)
4) Plurality recipient (categorical)
5) BMI recipient (continuous)
6) Smoking recipient (dichotomous)
7) Natural vs artificial cycle (dichotomous)
8) Ethnicity recipient (categorical)
9) Medical history of recipient related to hypertensive complications in pregnancy (dichotomous)
10) Family history of recipient related to hypertensive complications in pregnancy (dichotomous)
11) Age oocyte donor (continuous)
12) Sperm origin (father vs donor) (dichotomous)
13) Use of medication during pregnancy, specifically acetylsalicylic acid (dichotomous)

Data analysis

All statistical analyses will be performed using R.

Model development

A multivariate logistic regression model will be developed for the binary outcome hypertensive complications in pregnancy 'yes' and 'no'. The model will include the preselected candidate predictors.

As part of the secondary objectives, we aim to predict the risk on developing HELLP and time to development of PE. To achieve this, multivariate logistic regression models will be developed for the binary outcomes early PE (≤34 weeks) and HELLP 'yes' or 'no'. (43). Additionally, in order to predict the time of development of PE, a multivariate linear regression model will be developed for the continuous outcome gestation age at development of PE, with minimum of 20 weeks and maximum of 42 weeks)'. Possible non-linear associations between the continuous predictors and the outcome will be examined, using spline models. Potential multiplicative interaction will be explored by adding interaction terms to the model, and use Lasso regression for variable selection will be considered(47).

Model performance

Internal validation will be performed to assess model performance, discrimination and calibration will be determined. Discrimination describes the ability of the model to discriminate between events and non-events and will be evaluated with the area under the receiver curve (AUC). Calibration describes the relation between the observed risks within the population and the predicted risks and will be assessed by calibration plots, plotting the relation between the observed and predicted risks(48). Internal validation will be performed using the bootstrapping method with 100 bootstrap samples. The calibration slope from the bootstrapping procedure will be used as uniform shrinkage factor to calculate new regression coefficients to prevent overfitting(47). External validation will be performed using the 'DONation of Oocytes in Reproduction individual participant data' (DONOR IPD). The DONOR IPD database is currently being set up and will include at least 2301 women pregnant after OD and beyond 20 weeks of gestation(30). In this study original data from multiple studies will be combined into a single database is therefore suitable for model performance assessment and external validations(49, 50).

Handling of missing data

For peer review only - http://bmjopen.bmj.com/site/about/guidelines.xhtml

In case the amount of missing data in some of the predictors is substantial and cannot be ignored, a multiple imputation method will be applied(51, 52).

Patient and public involvement

During the development of the study protocol, Freya was consulted for input and advice. Freya is the Dutch society for patients with fertility problems and many patients who apply to OD are member of the association. Study information will be published on their website, and information on progress and results will be presented to patients during meetings, patient days and webinars.

For peer review only

For peer review only - http://bmjopen.bmj.com/site/about/guidelines.xhtml

Discussion

Each year, ten thousands of OD procedures are performed successfully worldwide (4-7). Although the numbers are rising due to delay of maternal age and increasing indications for OD, the method is accompanied with a high incidence of complications, in particular hypertensive disorders of pregnancy. The possibility to predict these complications could potentially aid in disease management and even prevention, yet current prognostic factors and prediction models are not developed, nor validated, for the OD population. With this project, we propose to develop the first prediction model on the risk of hypertensive complications in pregnancy in women considering OD. In clinical practice clinicians could use this developed prediction model to more accurately classify the woman as low or high risk for hypertensive complications in pregnancy, allowing for more personalized counseling for women considering OD. As a consequence this might facilitate in decision making for the patient. In the case the woman is already pregnant, knowledge on hypertensive complications risk might aid the clinician in early recognition of the disease and risk stratification during pregnancy, resulting in better health care management.

One fundamental strength of this project is that reporting will be in line with the TRIPOD guideline, aiming to reduce bias by enhancing transparency with regard to development, validation and updates of our model. (34, 36). Additionally, most PROBAST criteria will be met in this study, further mitigating bias.

Besides, available IPD (30) makes external validation possible. This will enable optimal performance evaluation and improve predictive accuracy, as populations independent of that used to develop the model will be used with different environmental, geographical, socioeconomic characteristics and different times of conception (30, 37).

It should be acknowledged that control of bias may be impeded as most data will be acquired from medical records. The prediction model will be developed and validated in a population that is already pregnant after OD, whereas the model is intended for facilitating pre-conceptional counseling. Although differences are expected to be small and insignificant, the prediction model might over- or underperform in the intended population group. Therefore, external validation in a population of women considering OD is warranted.

Furthermore, the present project also includes secondary objectives of predicting the severity of PE and its time of onset. The risk of developing severe PE is lower than the risk of hypertensive complication in OD. Additionally, the prediction of time onset of preeclampsia is only possible in patients that actually developed preeclampsia. Larger sample sizes are thus required for these objectives. Accordingly, development of these models will be undertaken during a subsequent phase.

Lastly, the predictor ‘fetal-maternal HLA-(mis)matches’ cannot be used as a candidate predictor in the model as it is unknown at time of proposed use of the model. The variable will be assessed to determine its individual prognostic value on the risk of developing hypertensive disease. In addition, the expected number of fetal-maternal mismatches could be calculated based on donor and paternal HLA-profiles. Still, the DONOR IPD dataset does not yet contain data on HLA-genotyping, thus the predictive value of fetal-maternal HLA-(mis)matches cannot be validated externally. To enable external validation in future, development of a database including information on fetal and maternal HLA-genotypes is required. We are currently working on a non-invasive method to perform antenatal HLA typing and calculation of mismatches. This will facilitate including the variable in future models and improve the accuracy of our predictions. Additionally, future studies should

For peer review only - http://bmjopen.bmj.com/site/about/guidelines.xhtml

carefully consider the financial implications of HLA typing as this may impact the feasibility of large-scale implementation and clinical practice. To conclude, this study aims to develop and externally validate the first prediction model on the risk of hypertensive complications in pregnancy in women considering OD. The possibility to predict the risk of hypertensive complications in pregnancy could potentially aid in disease management of women considering OD or in women pregnant after OD.

For peer review only

For peer review only - http://bmjopen.bmj.com/site/about/guidelines.xhtml

Ethics and Dissemination

The study will be conducted according to the principles of the Declaration of Helsinki. All patients will obtain written information of the study and in case of participation, the informed consent form should be signed prior to inclusion. This study is approved by the Medical Ethical Committee LDD (Leiden, Den Haag, Delft), with protocol number P16.048 and general assessment registration (ABR) number NL56308.058.16.. All clinical data and biomaterial is pseudonymised by assigning a unique code and will be collected in the data management system Castor. Only a minimum number of members of our project group will have access to this database. The results of our study will be disseminated via publications in gynecology related journals. Further, the results of our study might be presented at (international) conferences.

Ethics statements

Patient consent for publication: not applicable.

For peer review only

Abbreviations

AUC	Area Under the Curve
DONOR	DONation of Oocytes in Reproduction
HELLP	Heymolysis, Elevated Liver Enzymes and Low Platelets
HLA	Human Leukocyte Antigen
ICSI	Intracytoplasmic Sperm Injection
IPD	Individual Participant Data
IVF	In Vitro Fertilization
LUMC	Leiden University Medical Center
METC	Medical Ethics Committee
NC	Naturally conceived
PE	Preeclampsia
PROBAST	Prediction model Risk Of Bias Assessment Tool
ROB	Risk of Bias
TRIPOD	Transparent Reporting of a multivariable prediction model for Individual Prognosis Or Diagnosis

For peer review only

For peer review only - http://bmjopen.bmj.com/site/about/guidelines.xhtml

References

1. Lutjen P, Trounson A, Leeton J, et al. The establishment and maintenance of pregnancy using in vitro fertilization and embryo donation in a patient with primary ovarian failure. Nature. 1984;307(5947):174-5. doi: 10.1038/307174a0
2. Nederlandse Vereniging voor Klinische Embryologie (KLEM). Modelreglement Embryowet [online]. 2018. https://www.nvog.nl/wp-content/uploads/2020/10/Modelreglement-Embryowet-NVOG-en-KLEM-definitief-augustus-2018-aanpassing-15-oktober-2020.pdf
3. Nederlandse Vereniging voor Obstetrie en Gynaecologie (NVOG). STANDPUNT - Geassisteerde voortplanting met gedoneerde gameten en gedoneerde embryo's en draagmoederschap [online]. 2016. https://www.nvog.nl/wp-content/uploads/2020/06/Standpunt-Geassisteerde-voortplanting-met-gedoneerde-gameten-en-gedoneerde-embryo-juni-2016-addendum-12-6-2020.pdf.
4. Society For Assisted Reproductive Technology. National Summary Report [online]. 2018. https://www.sartcorsonline.com/rptCSR_PublicMultYear.aspx?reportingYear=2018.
5. Hogan RG, Wang AY, Li Z, et al. Oocyte donor age has a significant impact on oocyte recipients' cumulative live-birth rate: a population-based cohort study. Fertil Steril. 2019;112(4):724-30. doi: 10.1016/j.fertnstert.2019.05.012
6. Remohí J, Gartner B, Gallardo E, et al. Pregnancy and birth rates after oocyte donation. Fertil Steril. 1997;67(4):717-23. doi: 10.1016/s0015-0282(97)81372-6
7. Wyns C, De Geyter C, Calhaz-Jorge C, et al. ART in Europe, 2018: results generated from European registries by ESHRE†. Human Reproduction Open. 2022;2022(3). doi: 10.1093/hropen/hoac022
8. Savasi VM, Mandia L, Laoreti A, et al. Maternal and fetal outcomes in oocyte donation pregnancies. Hum Reprod Update. 2016;22(5):620-33. doi: 10.1093/humupd/dmw012
9. Bodri D, Nair S, Gill A et al. Shared motherhood IVF: high quality rates in a large study of treatments for lesbian couples using partner donated eggs. Reprod Biomed Online. 2018;36:130-6. doi: 10.1016/j.rbmo.2017.11.006
10. Storgaard M, Loft A, Bergh C, et al. Obstetric and neonatal complications in pregnancies conceived after oocyte donation: a systematic review and meta-analysis. Bjog. 2017;124(4):561-72. doi: 10.1111/1471-0528.14257
11. Van der Hoorn MP, van Egmond A, Swings G, et al. Differential immunoregulation in successful oocyte donation pregnancies compared with naturally conceived pregnancies. J Reprod Immunol. 2014;101-102:96-103. doi: 10.1016/j.jri.2013.08.002
12. Berntsen S, Larsen EC, la Cour Freiesleben N, et al. Pregnancy outcomes following oocyte donation. Best Pract Res Clin Obstet Gynaecol. 2021;70:81-91. doi: 10.1016/j.bpobgyn.2020.07.008
13. Berntsen S, Söderström-Anttila V, Wennerholm UB, et al. The health of children conceived by ART: 'the chicken or the egg?'. Hum Reprod Update. 2019;25(2):137-58. doi: 10.1093/humupd/dmz001
14. Masoudian P, Nasr A, de Nanassy J, et al. Oocyte donation pregnancies and the risk of preeclampsia or gestational hypertension: a systematic review and metaanalysis. Am J Obstet Gynecol. 2016;214(3):328-39. doi: 10.1016/j.ajog.2015.11.020

For peer review only - http://bmjopen.bmj.com/site/about/guidelines.xhtml

15. Levron Y, Dviri M, Segol I, et al. The 'immunologic theory' of preeclampsia revisited: a lesson from donor oocyte gestations. Am J Obstet Gynecol. 2014;211(4):383.e1-5. doi: 10.1016/j.ajog.2014.03.044
16. Blázquez A, García D, Rodríguez A, et al. Is oocyte donation a risk factor for preeclampsia? A systematic review and meta-analysis. J Assist Reprod Genet. 2016;33(7):855-63. doi: 10.1007/s10815-016-0701-9.
17. Moreno-Sepulveda J, Checa MA. Risk of adverse perinatal outcomes after oocyte donation: a systematic review and meta-analysis. J Assist Reprod Genet. 2019;36(10):2017-37. doi: 10.1007/s10815-019-01552-4
18. Jeve YB, Potdar N, Opoku A, et al. Donor oocyte conception and pregnancy complications: a systematic review and meta-analysis. Bjog. 2016;123(9):1471-80. doi: 10.1111/1471-0528.13910
19. Keukens A, van Wely M, van der Meulen C, et al. Pre-eclampsia in pregnancies resulting from oocyte donation, natural conception or IVF: a systematic review and meta-analysis. Hum Reprod. 2022;37(3):586-99. doi: 10.1093/humrep/deab267
20. World Health Organization. Maternal mortality: Key facts [online]. 2021. https://www.who.int/news-room/fact-sheets/detail/maternal-mortality
21. National Institute for Health and Care Excellence (UK): Clinical Guidelines. Hypertension in pregnancy: diagnosis and management [online]. 2019. https://www.nice.org.uk/guidance/ng133
22. Mol BWJ, Roberts CT, Thangaratinam S, et al. Pre-eclampsia. Lancet. 2016;387(10022):999-1011. doi: 10.1016/s0140-6736(15)00070-7
23. Patrícia Medeiros Falcão K, Pedrozo Campos Antunes T, do Nascimento Andrade Feitosa A, et al. Association between hypertension and quality of life in pregnancy. Hypertens Pregnancy. 2016;35(3):306-14. doi: 10.3109/10641955.2016.1143485
24. Van der Hoorn ML, Lashley EELO, Bianchi DW, et al. Clinical and immunologic aspects of egg donation pregnancies: a systematic review. Human Reproduction Update. 2010;16(6):704-12. doi: 10.1093/humupd/dmq017
25. Harrington K, Carpenter RG, Goldfrad C, et al. Transvaginal Doppler ultrasound of the uteroplacental circulation in the early prediction of pre-eclampsia and intrauterine growth retardation. Br J Obstet Gynaecol. 1997;104(6):674-81. doi: 10.1111/j.1471-0528.1997.tb11977.x
26. Snell KIE, Allotey J, Smuk M, et al. External validation of prognostic models predicting pre-eclampsia: individual participant data meta-analysis. BMC Med. 2020;18(1):302. doi: 10.1186/s12916-020-01766-9
27. Antwi E, Amoakoh-Coleman M, Vieira DL, et al. Systematic review of prediction models for gestational hypertension and preeclampsia. PLoS One. 2020;15(4):e0230955. doi: 10.1371/journal.pone.0230955
28. van Bentem K, Bos M, van der Keur C, et al. The development of preeclampsia in oocyte donation pregnancies is related to the number of fetal-maternal HLA class II mismatches. J Reprod Immunol. 2020;137:103074. doi: 10.1016/j.jri.2019.103074.
29. Lashley EELO, Haasnoot GW, Spruyt-Gerritse M, et al. Selective advantage of HLA matching in successful uncomplicated oocyte donation pregnancies. J Reprod Immunol. 2015;112:29-33. doi: 10.1016/j.jri.2015.05.006

For peer review only - http://bmjopen.bmj.com/site/about/guidelines.xhtml

30. van Bentem K, van der Hoorn ML, van Lith J, et al. Development of hypertensive complications in oocyte donation pregnancy: protocol for a systematic review and individual participant data meta-analysis (DONOR IPD). BMJ Open. 2022;12(7):e059594. doi: 10.1136/bmjopen-2021-059594
31. van Bentem K, Lashley EELO, Bos M, et al. Relating the number of human leucocytes antigen mismatches to pregnancy complications in oocyte donation pregnancies: study protocol for a prospective multicenter cohort study (DONOR study). BMJ Open. 2019;9(7):e027469. doi: 10.1136/bmjopen-2018-027469
32. Castor EDC (Electronic Data Capture). 2023. Available from: http://castoredc.com
33. Dalva B, Meral Beksac. HLA typing with sequence-specific oligonucleotide primed PCR (PCR-SSO)and use of the Luminex technology. Methods Mol Med. 2007;134:61-9. Doi: 10.1007/978-1-4614-9437-9_6
34. Moons KGM, Wolff RF, Riley RD, et al. PROBAST: A Tool to Assess Risk of Bias and Applicability of Prediction Model Studies: Explanation and Elaboration. Ann Intern Med. 2019;170(1):W1-w33. doi: 10.7326/m18-1377
35. van Bentem K, Bos M, van der Keur C, et al. Different immunoregulatory components at the decidua basalis of oocyte donation pregnancies. Hum Immunol. 2022;83(4):319-27. doi: 10.1016/j.humimm.2021.10.008.
36. Moons KG, Altman DG, Reitsma JB, et al. Transparent Reporting of a multivariable prediction model for Individual Prognosis or Diagnosis (TRIPOD): explanation and elaboration. Ann Intern Med. 2015;162(1):W1-73. doi: 10.7326/m14-0698
37. Collins GS, Reitsma JB, Altman DG, et al. Transparent reporting of a multivariable prediction model for individual prognosis or diagnosis (TRIPOD): the TRIPOD Statement. BMC Medicine. 2015;13(1):1. doi: 10.1186/s12916-014-0241-z
38. Riley RD, Ensor J, Snell KIE, et al. Calculating the sample size required for developing a clinical prediction model. BMJ. 2020;368:m441. doi: 10.1136/bmj.m441
39. Riley RD, Snell KIE, Ensor J, et al. Minimum sample size for developing a multivariable prediction model: PART II - binary and time-to-event outcomes. Stat Med. 2019;38(7):1276-96. doi: 10.1002/sim.7992
40. van Smeden M, Moons KG, de Groot JA, et al. Sample size for binary logistic prediction models: Beyond events per variable criteria. Stat Methods Med Res. 2019;28(8):2455-74. doi: 10.1177/0962280218784726
41. Ministry of Health Welfare and Sport (Netherlands). Annual Report 2020 of the Foundation Donor Data Artificial Fertilization. 2020:23.
42. Salama M, Isachenko V, Isachenko E, et al. Cross border reproductive care (CBRC): a growing global phenomenon with multidimensional implications (a systematic and critical review). J Assist Reprod Genet. 2018;35(7):1277-88. doi: 10.1007/s10815-018-1181-x
43. Magee, LA et al. The 2021 International Society for the Study of Hypertension in Pregnancy classification, diagnosis & management recommendations for international practice. Pregnancy Hypertens. 2022; 27: 148-169.
44. van Bentem K, Lashley EELO, van der Hoorn ML. Significance of specialised preconception counselling in oocyte donation pregnancy with prior history of postpartum eclampsia. BMJ Case Rep. 2020;13(11). doi: 10.1136/bcr-2020-235582
45. The FIGO Textbook of Pregnancy Hypertension. London: The Global Library of Women's Medicine; 2016.

For peer review only

For peer review only - http://bmjopen.bmj.com/site/about/guidelines.xhtml

46. Pimentel C, Solene D, Frédérique J, et al. What are the Predictive Factors for Preeclampsia in Oocyte Recipients? J Hum Reprod Sci. 2019;12(4):327-33. doi: 10.4103/jhrs.JHRS_43_19
47. Steyerberg EW. Clinical Prediction Models - A Practical Approach to Development, Validation, and Updating. Switzerland: Springer Nature Switzerland AG; 2019. 558 p.
48. Steyerberg EW, Vergouwe Y. Towards better clinical prediction models: seven steps for development and an ABCD for validation. Eur Heart J. 2014;35(29):1925-31. doi: 10.1093/eurheartj/ehu207
49. Riley RD, Lambert PC, Abo-Zaid G. Meta-analysis of individual participant data: rationale, conduct, and reporting. BMJ. 2010;340:c221. doi: 10.1136/bmj.c221
50. Stewart LA, Parmar MK. Meta-analysis of the literature or of individual patient data: is there a difference? Lancet. 1993;341(8842):418-22. doi: 10.1016/0140-6736(93)93004-k
51. White IR, Royston P, Wood AM. Multiple imputation using chained equations: Issues and guidance for practice. Stat Med. 2011;30(4):377-99. doi: 10.1002/sim.4067
52. van Ginkel JR, Linting M, Rippe RCA, et al. Rebutting Existing Misconceptions About Multiple Imputation as a Method for Handling Missing Data. J Pers Assess. 2020;102(3):297-308. doi: 10.1080/00223891.2018.1530680

For peer review only

For peer review only - http://bmjopen.bmj.com/site/about/guidelines.xhtml

Author's contributions

MLvdH and EL drafted the protocol, with SlC making substantial contributions to the epidemiological design of the work. EL and YL are the coordinating investigators in the LUMC and the Erasmus MC respectively. GL and VvdE wrote the protocol in accordance with the co-authors' contributions. GL is the corresponding author and submitted the manuscript. All authors gave final approval of the version to be published and agree to be accountable for all aspects of the work.

Funding statement

EL has received the ZonMw Clinical Fellows 2021 Grant, with project code 09032212110062.

Competing interests statement

EL has received the ZonMw Clinical Fellows 2021 Grant, with project code 09032212110062. YL has received honoraria for lectures and presentations by Merck© and Ferring©. Moreover, YL is supported by Gideon Richter© to attend the ORBIS program (a 3 year expert-led program). GL, VvdE, SlC and MLvdH have no competing interests.

For peer review only

Tables

Table 1. Baseline characteristics

Maternal characteristics	General<br>Date of birth, country of origin, ethnicity, socio-economic status, medical history, family history<br>Obstetric<br>Number of previous pregnancies, number of previous miscarriages/induced abortions/ectopic pregnancies or pregnancies of unknown location, indication for OD treatment<br>Laboratory<br>HLA-typing<br>Oocyte donation procedure<br>Age at time of embryo transfer, BMI at time of embryo transfer, smoking/drugs/alcohol use
Paternal characteristics	General<br>Date of birth, country of origin, ethnicity, medical history, family history<br>Sperm retrieval<br>Sperm from partner vs donor, age at time of sperm retrieval, BMI at time of sperm retrieval, smoking/alcohol/drugs use
Oocyte donor characteristics	Age at time of oocyte retrieval, country where oocyte retrieval took place, ethnicity<br>Laboratory[$]<br>HLA-typing
Procedure	Method used for fertilization (IVF/ICSI), transfer date, country where the transfer took place, transfer in natural or artificial cycle, use of medication (recipient), fresh or cryopreserved transfer of embryo, number of transferred embryos, kind of pregnancy (oocyte donation, embryo donation, or surrogate pregnancy)
Pregnancy	Highest systolic- and diastolic blood pressure, proteinuria, diagnosis of PIH/PE/HELLP, use of salicylic acid during pregnancy, addition or change of medication during pregnancy
Delivery and neonatal characteristics	Place of birth (hospital vs home), mode of delivery, medication use during labour, gestational age, singleton or multiple, gender, birthweight, live birth (yes/no)

[$]In case the donor is known and wants to participate, one sample of peripheral blood could be obtained in order to perform HLA-typing.

For peer review only

For peer review only - http://bmjopen.bmj.com/site/about/guidelines.xhtml

TRIPOD Checklist: Prediction Model Development

Section/Topic	Item	Checklist Item	Page
**Title and abstract**			
Title	1	Identify the study as developing and/or validating a multivariable prediction model, the target population, and the outcome to be predicted.	1
Abstract	2	Provide a summary of objectives, study design, setting, participants, sample size, predictors, outcome, statistical analysis, results, and conclusions.	2
**Introduction**			
Background and objectives	3a	Explain the medical context (including whether diagnostic or prognostic) and rationale for developing or validating the multivariable prediction model, including references to existing models.	3
	3b	Specify the objectives, including whether the study describes the development or validation of the model or both.	4
**Methods**			
Source of data	4a	Describe the study design or source of data (e.g., randomized trial, cohort, or registry data), separately for the development and validation data sets, if applicable.	5
	4b	Specify the key study dates, including start of accrual; end of accrual; and, if applicable, end of follow-up.	5
Participants	5a	Specify key elements of the study setting (e.g., primary care, secondary care, general population) including number and location of centres.	5
	5b	Describe eligibility criteria for participants.	5
	5c	Give details of treatments received, if relevant.	n.a.
Outcome	6a	Clearly define the outcome that is predicted by the prediction model, including how and when assessed.	7
	6b	Report any actions to blind assessment of the outcome to be predicted.	n.a.
Predictors	7a	Clearly define all predictors used in developing or validating the multivariable prediction model, including how and when they were measured.	7-8
	7b	Report any actions to blind assessment of predictors for the outcome and other predictors.	n.a.
Sample size	8	Explain how the study size was arrived at.	7
Missing data	9	Describe how missing data were handled (e.g., complete-case analysis, single imputation, multiple imputation) with details of any imputation method.	8
Statistical analysis methods	10a	Describe how predictors were handled in the analyses.	n.a.
	10b	Specify type of model, all model-building procedures (including any predictor selection), and method for internal validation.	8
	10d	Specify all measures used to assess model performance and, if relevant, to compare multiple models.	8
Risk groups	11	Provide details on how risk groups were created, if done.	n.a.
**Results**			
Participants	13a	Describe the flow of participants through the study, including the number of participants with and without the outcome and, if applicable, a summary of the follow-up time. A diagram may be helpful.	n.a.
	13b	Describe the characteristics of the participants (basic demographics, clinical features, available predictors), including the number of participants with missing data for predictors and outcome.	n.a.
Model development	14a	Specify the number of participants and outcome events in each analysis.	n.a.
	14b	If done, report the unadjusted association between each candidate predictor and outcome.	n.a.
Model specification	15a	Present the full prediction model to allow predictions for individuals (i.e., all regression coefficients, and model intercept or baseline survival at a given time point).	n.a.
	15b	Explain how to the use the prediction model.	n.a.
Model performance	16	Report performance measures (with CIs) for the prediction model.	n.a.
**Discussion**			
Limitations	18	Discuss any limitations of the study (such as nonrepresentative sample, few events per predictor, missing data).	9
Interpretation	19b	Give an overall interpretation of the results, considering objectives, limitations, and results from similar studies, and other relevant evidence.	n.a.
Implications	20	Discuss the potential clinical use of the model and implications for future research.	9
**Other information**			
Supplementary information	21	Provide information about the availability of supplementary resources, such as study protocol, Web calculator, and data sets.	n.a.
Funding	22	Give the source of funding and the role of the funders for the present study.	17

We recommend using the TRIPOD Checklist in conjunction with the TRIPOD Explanation and Elaboration document.

For peer review only - http://bmjopen.bmj.com/site/about/guidelines.xhtml

STROBE Statement—Checklist of items that should be included in reports of ***cohort studies***

	Item No	Recommendation	Page No
**Title and abstract**	1	(*a*) Indicate the study's design with a commonly used term in the title or the abstract	1
		(*b*) Provide in the abstract an informative and balanced summary of what was done and what was found	2
**Introduction**			
Background/rationale	2	Explain the scientific background and rationale for the investigation being reported	3
Objectives	3	State specific objectives, including any prespecified hypotheses	4
**Methods**			
Study design	4	Present key elements of study design early in the paper	5
Setting	5	Describe the setting, locations, and relevant dates, including periods of recruitment, exposure, follow-up, and data collection	5
Participants	6	(*a*) Give the eligibility criteria, and the sources and methods of selection of participants. Describe methods of follow-up	5
		(*b*) For matched studies, give matching criteria and number of exposed and unexposed	n.a.
Variables	7	Clearly define all outcomes, exposures, predictors, potential confounders, and effect modifiers. Give diagnostic criteria, if applicable	7-8
Data sources/ measurement	8*	For each variable of interest, give sources of data and details of methods of assessment (measurement). Describe comparability of assessment methods if there is more than one group	5
Bias	9	Describe any efforts to address potential sources of bias	6
Study size	10	Explain how the study size was arrived at	7
Quantitative variables	11	Explain how quantitative variables were handled in the analyses. If applicable, describe which groupings were chosen and why	n.a.
Statistical methods	12	(*a*) Describe all statistical methods, including those used to control for confounding	7-8
		(*b*) Describe any methods used to examine subgroups and interactions	n.a.
		(*c*) Explain how missing data were addressed	8
		(*d*) If applicable, explain how loss to follow-up was addressed	n.a.
		(*e*) Describe any sensitivity analyses	n.a.
**Results**			
Participants	13*	(a) Report numbers of individuals at each stage of study—eg numbers potentially eligible, examined for eligibility, confirmed eligible, included in the study, completing follow-up, and analysed	n.a.
		(b) Give reasons for non-participation at each stage	n.a.
		(c) Consider use of a flow diagram	n.a.
Descriptive data	14*	(a) Give characteristics of study participants (eg demographic, clinical, social) and information on exposures and potential confounders	n.a.
		(b) Indicate number of participants with missing data for each variable of interest	n.a.
		(c) Summarise follow-up time (eg, average and total amount)	n.a.
Outcome data	15*	Report numbers of outcome events or summary measures over time	n.a.

For peer review only

For peer review only - http://bmjopen.bmj.com/site/about/guidelines.xhtml

Main results	16	(*a*) Give unadjusted estimates and, if applicable, confounder-adjusted estimates and their precision (eg, 95% confidence interval). Make clear which confounders were adjusted for and why they were included	n.a.
		(*b*) Report category boundaries when continuous variables were categorized	n.a.
		(*c*) If relevant, consider translating estimates of relative risk into absolute risk for a meaningful time period	n.a.
Other analyses	17	Report other analyses done—eg analyses of subgroups and interactions, and sensitivity analyses	n.a.
**Discussion**			
Key results	18	Summarise key results with reference to study objectives	n.a.
Limitations	19	Discuss limitations of the study, taking into account sources of potential bias or imprecision. Discuss both direction and magnitude of any potential bias	9
Interpretation	20	Give a cautious overall interpretation of results considering objectives, limitations, multiplicity of analyses, results from similar studies, and other relevant evidence	n.a.
Generalisability	21	Discuss the generalisability (external validity) of the study results	9
**Other information**			
Funding	22	Give the source of funding and the role of the funders for the present study and, if applicable, for the original study on which the present article is based	17

*Give information separately for exposed and unexposed groups.

Note: An Explanation and Elaboration article discusses each checklist item and gives methodological background and published examples of transparent reporting. The STROBE checklist is best used in conjunction with this article (freely available on the Web sites of PLoS Medicine at http://www.plosmedicine.org/, Annals of Internal Medicine at http://www.annals.org/, and Epidemiology at http://www.epidem.com/). Information on the STROBE Initiative is available at http://www.strobe-statement.org.

For peer review only

For peer review only - http://bmjopen.bmj.com/site/about/guidelines.xhtml
